# Genome-wide identification, phylogenetic classification of histone acetyltransferase genes, and their expression analysis in sugar beet (*Beta vulgaris* L.) under salt stress

**DOI:** 10.1007/s00425-024-04361-x

**Published:** 2024-03-06

**Authors:** Seher Yolcu, Monika Skorupa, Mehmet Emin Uras, Justyna Mazur, Ibrahim Ilker Ozyiğit

**Affiliations:** 1 Faculty of Engineering and Natural Sciences, Sabanci University, 34956 Istanbul, Türkiye; 2 Faculty of Biological and Veterinary Sciences, Nicolaus Copernicus University, 87-100 Torun, Poland; 3 Centre for Modern Interdisciplinary Technologies, Nicolaus Copernicus University, 87-100 Torun, Poland; 4 Faculty of Arts and Sciences, Department of Molecular Biology and Genetics, Haliç University, 34060 Istanbul, Türkiye; 5 Faculty of Science, Department of Biology, Marmara University, 34722 Istanbul, Türkiye

**Keywords:** *Beta vulgaris*, Genome-wide analysis, Histone acetyltransferase (HAT), Salt stress, Sugar beet

## Abstract

**Main conclusion:**

This study identified seven histone acetyltransferase-encoding genes (*HAT*s) from *Beta vulgaris* L. (sugar beet) genome through bioinformatics tools and analyzed their expression profiles under salt stress. Sugar beet HATs are phylogenetically divided into four families: GNAT, MYST, CBP, and TAFII250. The *BvHAT* genes were differentially transcribed in leaves, stems, and roots of *B. vulgaris* salt-resistant (Casino) and -sensitive (Bravo) cultivars under salt stress.

**Abstract:**

Histone acetylation is regulated by histone acetyltransferases (HATs), which catalyze ɛ-amino bond formation between lysine residues and acetyl groups with a cofactor, acetyl-CoA. Even though the HATs are known to participate in stress response and development in model plants, little is known about the functions of HATs in crops. In sugar beet (*Beta vulgaris* L.), they have not yet been identified and characterized. Here, an in silico analysis of the *HAT* gene family in sugar beet was performed, and their expression patterns in leaves, stems, and roots of *B. vulgaris* were analyzed under salt stress. Salt-resistant (Casino) and -sensitive (Bravo) beet cultivars were used for gene expression assays. Seven *HATs* were identified from sugar beet genome, and named *BvHAG1, BvHAG2, BvHAG3, BvHAG4, BvHAC1, BvHAC2,* and *BvHAF1*. The HAT proteins were divided into 4 groups including MYST, GNAT (GCN5, HAT1, ELP3), CBP and TAFII250. Analysis of *cis-*acting elements indicated that the *BvHAT* genes might be involved in hormonal regulation, light response, plant development, and abiotic stress response. The *BvHAT* genes were differentially expressed in leaves, stems, and roots under control and 300 mM NaCl. In roots of *B. vulgaris* cv. Bravo, the *BvHAG1, BvHAG2, BvHAG4, BvHAF1*, and *BvHAC1* genes were dramatically expressed after 7 and 14 days of salt stress. Interestingly, the *BvHAC2* gene was not expressed under both control and stress conditions. However, the expression of *BvHAG2, BvHAG3, BvHAG4, BvHAC1, BvHAC2* genes showed a significant increase in response to salt stress in the roots of cv. Casino. This study provides new insights into the potential roles of histone acetyltransferases in sugar beet.

**Supplementary Information:**

The online version contains supplementary material available at 10.1007/s00425-024-04361-x.

## Introduction

Among distinct histone modifications, histone acetylation is the well-studied one that plays a considerable role in regulation of gene expression by decondensation of chromatin (Strahl and Allis 2000; Kim et al. 2010; Pandey et al. 2002). Transfer of an acetyl group (CH_3_CO) to the lysine residue of histone N-terminal tails decreases the net positive charge of histones and makes the DNA accessible for transcriptional activation (Sterner and Berger 2000; Shahbazian and Grunstein 2007). Histone acetylation and deacetylation are catalyzed by histone acetyltransferases (HATs) and histone deacetylases (HDACs) (Kouzarides 2007; Kim et al. 2015). HATs perform acetylation of histones at the promoter regions of genes (Kouzarides 2007). In eukaryotes, there are five HAT families; general control nondepressible 5 (GCN5)-related N-acetyltransferase (GNAT); MYST-MOZ, Ybf2/Sas3, Sas2, and Tip60; CREB-binding protein (CBP); and TFII250-TATA binding protein associated factors and the nuclear hormone-related HATs (Sterner and Berger 2000; Pandey et al. 2002). Four histone acetyltransferase families including GNAT (GCN5, ELP3 and HAT1), MYST (HAG4 and HAG5), p300/CBP (HAC1, HAC2, HAC4, HAC5, and HAC12) and TAFII250 (HAF1 and HAF2) are present in *Arabidopsis* genome (Pandey et al. 2002). A total of 12, 8, 32, 7, 6, 31, 14, 24, and 30 HAT-encoding genes were characterized in different plant species, such as *Arabidopsis* (Pandey et al. 2002), *Oryza sativa* (rice) (Liu et al. 2012), *Solanum lycopersicum* (tomato) (Aiese Cigliano et al. 2013; Hawar et al. 2021), *Vitis vinifera* (grapevine) (Aquea et al. 2010), *Litchi chinensis* (litchi) (Peng et al. 2017), *Triticum aestivum* (wheat) (Gao et al. 2021), *Citrus sinensis* (citrus) (Shu et al. 2021), *Setaria italica* (foxtail millet) (Xing et al. 2022), and *Capsicum annuum* (pepper) (Cai et al. 2022), respectively. HATs were reported to be involved in development, root stem cell niche maintenance, fruit development, flowering, and abiotic/biotic stress response in plants (Gao et al. 2021; Cai et al. 2022). Two *MYST* genes, *HAM1* and *HAM2* in *Arabidopsis* are functionally redundant gene pairs, *Arabidopsis ham1 ham2* double mutants showed severe defects in the male and female gametophyte development (Latrasse et al. 2008).

Salinity in drought or semiarid regions is one of the major environmental stresses that limits plant growth and production (Allakhverdiev et al. 2000). In plants, environmental stresses bring about epigenetic alterations, including DNA methylation, histone modifications, and ATP-dependent chromatin remodeling (Yuan et al. 2013; Chinnusamy et al. 2008). Chromatin modifications and small RNAs play a key role in gene regulation depending on the tissue, plant species/cultivar, organelle, and developmental stage (Sahu et al. 2013; Madlung and Comai 2004). Less is known about the involvement of epigenetic alterations on plant metabolism and physiology when compared to mammals. Genome-wide studies were performed to discover *HAT* genes in crop species, and their expression profiles varied in different tissues, developmental stages, and according to type and duration of stress (Cai et al. 2022; Shu et al. 2021; Xing et al. 2022; Gao et al. 2021; Peng et al. 2017). For example, *HAT* genes in *Setaria italica* were found to respond to different stresses, such as salt, drought, low nitrogen, and low phosphorus (Xing et al. 2022). The transcription abundances of *Capsicum annuum HATs (CaHAM1, CaHAG7, CaHA14, CaHAG5,* and *CaHAC4)* were upregulated at the early development stages of fruits, while the others were transcribed at the late developmental stages, suggesting the involvement of HATs in the regulation of fruit ripening (Cai et al. 2022). In a recent study, drought stress led to increase the expression of *Citrus sinensis HAT* genes *(CsHAT6, 13,* and *14)*, and decrease *CsHAT5* and *CsHAT8* transcription levels (Shu et al. 2021).

Sugar beet (*Beta vulgaris* L.) which belongs to the Amaranthaceae family, is a diploid (2*n* = 18) crop (Dohm et al. 2014), and used for production of the sugar, bioethanol, animal feed, and raw materials around the world (Hoffmann 2010; Yolcu et al. 2022; Yu et al. 2020). In addition to its commercial importance, sugar beet is known as a salt- and drought-tolerant crop plant (Wedeking et al. 2016), which can grow in calcareous, saline, alkaline, poor, and fertile soils (Hussein et al. 2019). Even though the sugar beet is sensitive to salt at the germination and seedling stages (Bor et al. 2003; Dunajska-Ordak et al. 2014), different beet varieties from distinct locations can withstand salt stress at these developmental stages. For example, among three Portuguese wild beet varieties (Comporta, Oeiras and Vaiamonte), the Comporta was able to initiate and maintain radicle emergence under high salt concentrations (Pinheiro et al. 2018). Genetic and physical maps depending on Single-Nucleotide Polymorphism were generated and transcriptomic studies were carried out to find out metabolic pathways and stress response genes in sugar beet (Lv et al. 2018; Geng et al. 2019; Dohm et al. 2011). Additionally, genome-wide identification of *B-box (BBX)* genes, *BRASSINAZOLE-RESISTANT (BZR)* family genes, and *high affinity K*^+^*-transporter (HAK)* genes in sugar beet has been carried out by in silico methods (Wang et al. 2019; Yang et al. 2022; Song et al. 2023). However, there are few research articles regarding the impacts of epigenetic modifications on gene regulation in *B. vulgaris* under salinity stress (Yolcu et al. 2016; Skorupa et al. 2021). In a recent study, eight *RPD3/HDA1* family members of histone deacetylase (HDAC)-encoding genes in *B. vulgaris* have been identified and characterized through bioinformatics tools and databases (Yu et al. 2023). The transcription levels of *BvHDACs* were altered in response to salt (300 mM NaCl), drought (6% PEG-6000) and cold (4 °C) stresses (Yu et al. 2023). Therefore, identification and characterization of gene families are required for developing highly stress-tolerant sugar beet varieties, which is important for sugar beet growth in the soils unsuitable for agriculture due to poor soil quality (Zhang et al. 2021). In sugar beet, except for *RPD3/HDA1-type HDAC* gene family (Yu et al. 2023), no histone modifier proteins have been previously identified by bioinformatics tools or wet-lab techniques.

This study aims to identify and characterize the HAT-encoding gene family in sugar beet through bioinformatics tools and databases. The study investigates their physical and chemical properties, phylogenetic relationships, subcellular localization, chromosomal distribution, syntenic relationship, conserved motifs, gene structure, protein 3D structures, and *cis-*acting regulatory elements in promoter regions. Besides, our study analyzed how these genes responded to salt stress in different tissues, such as stems, roots, and leaves of salt-resistant (Casino) and sensitive (Bravo) sugar beet genotypes with a comparative approach. Hence, the study’s findings will serve as an initial step toward future research on the epigenetic regulation of responses to abiotic stress.

## Materials and methods

### Identification of *HAT *genes in *B. vulgaris*

A total of 12 HAT protein sequences in *Arabidopsis* were retrieved from The Arabidopsis Information Resource (TAIR; https://www.arabidopsis.org/), and then used to search BvHATs with the BLASTP tool using sugar beet genome (*Beta vulgaris* ssp. *vulgaris* EL10.2_2, Phytozome genome ID: 782, NCBI taxonomy ID: 3555) in Phytozome (version 13; https://phytozome.jgi.doe.gov/pz/portal.html). All homologous protein sequences of the BvHAT candidates are accepted if they have sequence identity with *Arabidopsis* HAT proteins more than 55% and *e* < 10^–10^. Acetyltrasf_1, BROMO, Hat1-N, Elp3, CHROMO, ZnF-C2H2, ZnF_ZZ, MOZ-SAS, ZnF-TAZ, PHD, TBP-binding, and UBQ domains of *B. vulgaris* candidate proteins were confirmed by HMMER-based SMART (http://smart.embl-heidelberg.de/) (Letunic et al. 2012) and NCBI CDD (https://www.ncbi.nlm.nih.gov/cdd/) databases. Seven *HAT* genes are named *BvHAG1, BvHAG2, BvHAG3, BvHAG4, BvHAC1, BvHAC2,* and *BvHAF1* based on their chromosomal positions and HAT classification of plants. Physicochemical properties of seven BvHAT proteins, such as isoelectric point (pI), theoretical molecular weight (MW), and GRAVY, were predicted online at ExPASy server (https://web.expasy.org/protparam/) (Gasteiger et al. 2005).

### Prediction of subcellular localization

Subcellular localization predictions of HAT proteins were performed using two online predictors including CELLO server (http://cello.life.nctu.edu.tw/) (Yu et al. 2006), and WoLFPSORT (https://wolfpsort.hgc.jp/) (Nakai and Horton 1999; Horton et al. 2007).

### Phylogenetic analysis

To understand the evolutionary relationship of 7 BvHATs with other HATs, a total of 44 HAT protein sequences from different plant species, such as tomato, rice, and *Arabidopsis*, were retrieved from TAIR, National Center for Biotechnology Information (NCBI) and Ensembl Plants (https://plants.ensembl.org/index.html) (Bolser et al. 2017). Sugar beet HAT amino acid sequences were aligned using Clustal W with default parameters. Phylogenetic tree was constructed by MEGA11 (https://www.megasoftware.net/history.php) using the maximum likelihood statistical method, with 1000 bootstrap replicates, Poisson substitution model, and Nearest-Neighbor-Interchange as ML heuristic method (Tamura et al. 2021).

### Conserved motifs and the structure of *BvHAT* genes

Conserved motifs of the BvHATs were determined using the MEME tool (http://meme-suite.org/tools/meme) with the following parameters: the maximum number of motifs is 20 (Bailey and Elkan 1994). Gene Structure Display Server (GSDS) (http://gsds.gao-lab.org/) (Bo et al. 2015) was used to analyze the exon–intron organizations of the *BvHAT* genes.

### Chromosomal distribution and Ka/Ks ratio

The physical positions of the *BvHAT* genes along each chromosome were retrieved from the sugar beet genome (Ensembl Plants) and the chromosomal distribution graph was drawn by Mapgene2chrom 2.1 (MG2C v2.1) online tool (http://mg2c.iask.in/mg2c_v2.1/) (Chao et al. 2021; Jiangtao et al. 2015).

To indicate selective pressures on *BvHAT* genes, the ratios of non-synonymous to synonymous substitutions (Ka/Ks) of gene pairs were calculated by an online Ka Ks calculation tool (https://services.cbu.uib.no/tools/kaks). Divergence time was calculated using synonymous mutation rate of substitutions per synonymous site per year (T, MYA). Ka/Ks ratio was used to find the ratio between the non-synonymous substitution rate (Ka) and the synonymous substitution rate (Ks) of *BvHAT* genes, and divergence time was calculated using the following formula: *T* = Ks/2*λ*. The value of Ka/Ks ratio lower than 1 represents negative or stabilizing selection.

### Synteny analysis

Genomic synteny was comparatively performed to investigate the evolutionary relationship between sugar beet, rice, tomato, and *Arabidopsis* HAT proteins using the circoletto program (Circos) (tools.bat.inspire.org/circoletto/) (Krzywinski et al. 2009). Score/max ratio was used coloring with blue ≤ 0.25, green  ≤  0.50, orange  ≤  0.75, red > 0.75. Seven HAT protein sequences from *B. vulgaris* and forty-four HATs from *Arabidopsis*, rice, and tomato in FASTA format were included into query and database file, respectively.

### Protein 3D structure analysis

The amino acid sequences obtained from the Phytozome database were used to predict 3D structures for all identified BvHAT proteins. 3D modeling was performed in the Protein Homology/Analogy Recognition Engine V 2.0 (Phyre^2^) server using intensive mood (http://www.sbg.bio.ic.ac.uk/phyre2/) (Kelley et al. 2015). Validation of the 3D structures was performed by evaluating Ramachandran plots using the MolProbity database (Williams et al. 2018) and the SwissModel database structure assessment tool (Waterhouse et al. 2018). For the prediction of secondary structural elements of BvHATs, the SOPMA server was used (https://npsa-prabi.ibcp.fr) (Geourjon and Deléage 1995).

### Analysis of *cis*-acting regulatory elements

The sequences 1500 bp upstream of the transcription start site were extracted from the sugar beet genome using the Phytozome database. The numbers and the types of *cis-*elements were predicted by PlantCARE software (http://bioinformatics.psb.ugent.be/webtools/plantcare/html/) (Lescot et al. 2002).

### Plant materials for gene expression analysis

Two sugar beet cultivars, *Beta vulgaris* subsp. *vulgaris* L. cv. Bravo and *Beta vulgaris* subsp. *vulgaris* L. cv. Casino were used as plant materials. Sugar beet seeds were obtained from Greater Poland Sugar Beet Breeding–WHBC (Poznań, Poland). The seeds (5 per pots) were sown into pots filled with sand and vermiculite (1/1, *v*/*v*) and plants were watered regularly with half-strength Hoagland solution (Hoagland and Arnon 1950). Plants were cultured for four weeks in a growth chamber with a photoperiod of 16 h of light and 8 h of darkness with standard irradiation of 30 ± 5 µmol m^−2^ s^−1^, provided by T8 15 W 6500 K “Daylight” tubes (POLAMP, Ełk, Poland). The temperature regime was 25 °C during the day and 18 °C at night.

### Exposure of plants to salt stress

Salt treatment was started when the first pairs of mature leaves were fully developed. Over the first 7 days of treatment, plants were watered in two-day-long intervals with half-strength Hoagland solution supplemented with increasing concentrations of NaCl, until the final concentration of 300 mM NaCl was reached (first day of treatment–50 mM NaCl, third day–100 mM NaCl, fifth day–200 mM NaCl, seventh day–300 mM NaCl). Untreated controls were watered with a NaCl-free medium. Plants were watered with 200 mL of solution per 2 L of sand/vermiculite mixture. Materials (stems, roots, and leaves) for analysis were collected on the 7th, 14th and 21st day of stress treatment.

### Gene expression analysis

Total RNA was isolated from plant tissue using a GeneMATRIX Universal RNA Purification Kit (EURx) and digested using DNase I (Thermo Scientific) according to the manufacturer’s instructions. First-strand cDNA was synthesized from 1 μg of total RNA using random hexamers and First Strand cDNA Synthesis Kit for RT-PCR (Roche), following the manufacturer’s instructions. The gene-specific primers for qPCR were designed with Primer3Plus software. The β-actin gene was used as a reference. To determine the PCR efficiencies, standard curves for both target and control genes were obtained using a series of cDNA dilutions as a template. The RT-qPCR was performed on a LightCycler^®^ 480 using LightCycler^®^ 480 SYBR Green I Master, following the manufacturer’s protocol (Roche). Three independent biological replicates and three technical replicates were analyzed. Relative levels of gene expression were calculated according to the 2^−∆∆C(t)^ method (Livak and Schmittgen 2001). A list of the PCR primers used for the experiments is provided in Table S1.

### Data analysis

The statistical significance of differences between control samples and those from tissues treated with salt stress was determined using one-way ANOVA followed by Tukey’s test in SigmaPlot 14.5 (Systat Software). Differences of *p* < 0.05 were considered significant. The mean and the standard deviation were calculated. Error bars shown in all figures represented the standard deviation calculated from three repetitions of each experiment.

## Results

### Identification of *HAT* genes in *B. vulgaris*

The protein sequences of 12 HATs in *Arabidopsis* were obtained from TAIR, and Phytozome 13, and then these queries were used to search HAT proteins of *B. vulgaris* through BLASTP. A total of seven HATs in *B. vulgaris* were detected (BvHAG1, BvHAG2, BvHAG3, BvHAG4, BvHAC1, BvHAC2, and BvHAF1) and named according to the plant HAT families and their positions on chromosomes (Table 1). The *B. vulgaris* HATs were classified according to the protein motifs found in *Arabidopsis* HATs. For instance, three proteins BvHAG2, BvHAG3, and BvHAG4 consisted of Acetyltransf_1 motif, and they had similar amino acid lengths. CBP family members, BvHAC1 and BvHAC2 contained 3 motifs, such as PHD, ZnF_ZZ, and ZnF_TAF, specifically found in *Arabidopsis* CBP members. The physicochemical properties of *HAT* genes and HAT proteins, such as chromosome location, strand, CDS (bp), amino acid length (aa), molecular weight (MW), isoelectric points (pI), and grand average of hydropathicity (GRAVY), were extracted from an online tool, Expasy ProtParam (Table 1). The HAT protein lengths ranged from 432 (*BvHAG3*) to 1908 (*BvHAF1*) aa. The predicted MWs were between 48.72 and 215.35 kDa, and the pI was 5.31–8.73.

**Table 1:** The physicochemical properties of seven *HAT* genes in *B. vulgaris* that were computed by Expasy ProtParam tool

Sequence ID	Gene name	HAT group	Chromosome location	Strand	CDS (bp)	Length (aa)	MW (kDa)	pI	GRAVY
Bevul.1G214800	*HAG1*	MYST	Chr1:63,743,451–63,751,102	Reverse	1344	447	51.84	6.37	− 0.619
Bevul.2G218200	*HAC1*	CBP	Chr2:53,307,479–53318523	Reverse	4347	1448	161.69	6.40	− 0.409
Bevul.3G111900	*HAF1*	TAFII250	Chr3:16,418,854–16,446,206	Forward	5727	1908	215.35	5.58	− 0.713
Bevul.5G241300	*HAG2*	GNAT-GCN5	Chr5:65,462,103–65469074	Reverse	1371	456	49.80	6.60	− 0.665
Bevul.7G116000	*HAG3*	GNAT-HAT1	Chr7:38,354,718–38,357,990	Reverse	1299	432	48.72	5.31	− 0.220
Bevul.7G145700	*HAG4*	GNAT-ELP3	Chr7:48,102,439–48107621	Reverse	1701	566	63.54	8.73	− 0.338
Bevul.7G147900	*HAC2*	CBP	Chr7:48,509,834–48522625	Forward	5136	1711	191.59	8.53	− 0.708

The computed parameters consist of the molecular weight (MW), theoretical pI, amino acid length, and grand average of hydropathicity (GRAVY)

### Subcellular localization prediction

Bioinformatics tools have been widely used to predict subcellular locations of proteins that can guide researchers in designing wet-lab studies to estimate the certainty of predictions (Dönnes and Höglund 2004). Our study includes only in silico approach. Online predictors (cello-life, and WoLFPSORT) were used to predict subcellular localization of BvHATs, which are presented in Table 2. Except for BvHAG4, almost all HAT proteins were found to be localized in nucleus according to cello-life tool. BvHAG3 and BvHAG4 were present in cytoplasm/nucleus, and mitochondrion, respectively. Consistent with cello-life results, WoLF PSORT also showed the nuclear localization of BvHAC1, BvHAC2, and BvHAF1 with high frequencies (Table 2). The subcellular localizations of BvHAG2 and BvHAG4 were found in the chloroplast and cytosol, respectively. Interestingly, BvHAG3 was assumed to be present in endoplasmic reticulum. Even though the prediction tools were shown the nuclear localizations of BvHATs, it is important to note that the BvHAT proteins located in different cellular compartments, such as cytosol, nucleus, endoplasmic reticulum, and chloroplast.

**Table 2:** Predicted subcellular localization of HAT proteins in *B. vulgaris*

Protein	Subcellular localization	
	Cello-life	WoLF PSORT
HAG1	Nuclear	cyto (6.5), cyto_nucl (6.5)
HAC1	Nuclear	nucl (12), cyto (1)
HAF1	Nuclear	nucl (11), cyto (3)
HAG2	Nuclear	chlo (12), nucl (1)
HAG3	Cytoplasmic/nuclear	ER (6), cyto (3)
HAG4	Mitochondrial	cyto (6), mito (4)
HAC2	Nuclear	nucl (14)

Two online prediction tools, such as cello-life and WoLFPSORT, were used to examine the subcellular localization of *B. vulgaris* HAT proteins (*cyto: cytosol, nucl: nucleus, plas: plasma membrane, ER: endoplasmic reticulum, mito: mitochondrion, chlo: chloroplast*)

### Phylogenetic relationships

**Fig. 1:**
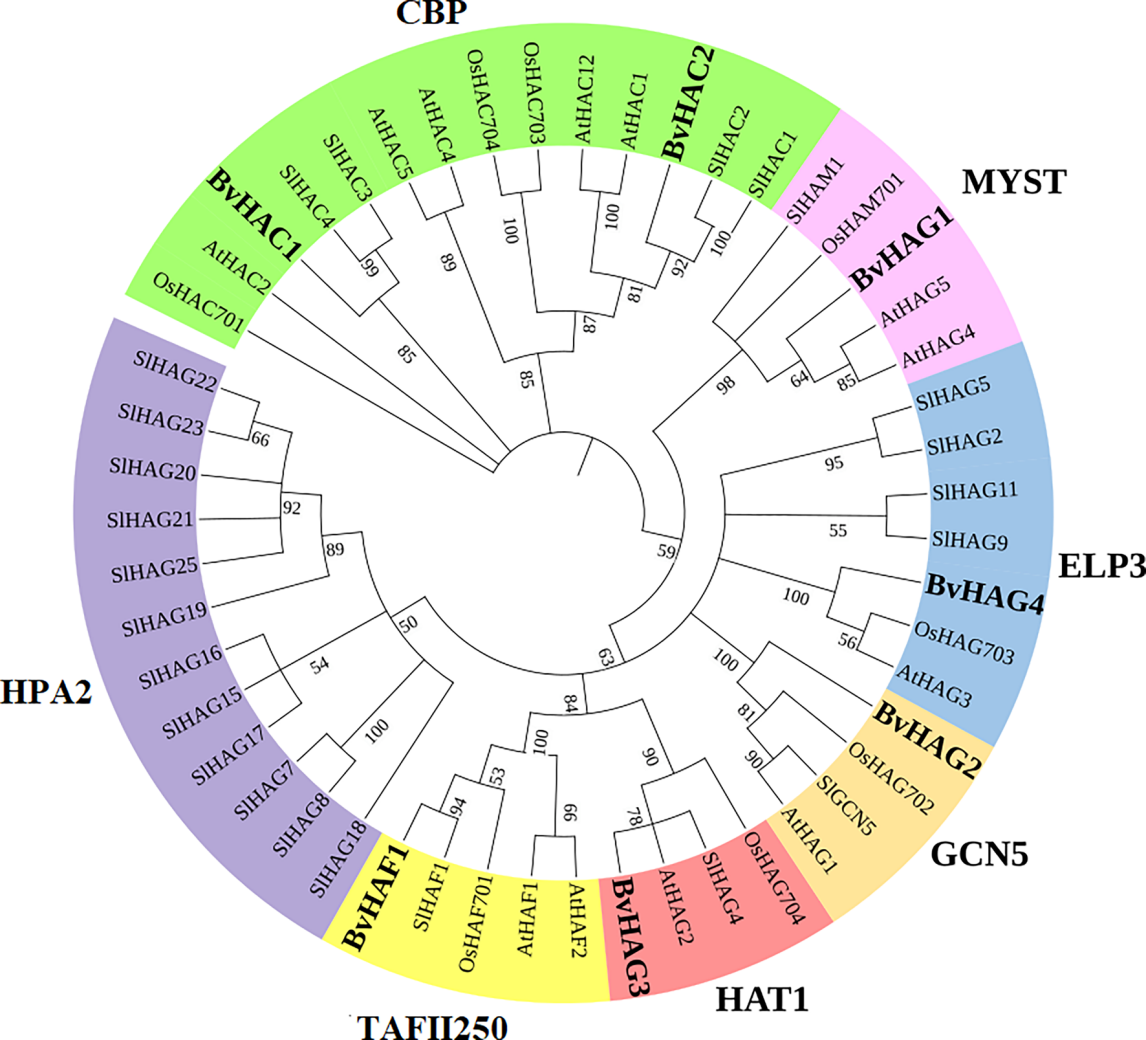
Phylogenetic tree of 51 HAT proteins in different plant species, including *B. vulgaris (Bv), Arabidopsis thaliana (At), Solanum lycopersicum (SI),* and *Oryza sativa (Os)*. Maximum Likelihood method and Poisson correction model were used to generate the phylogenetic tree (1000 bootstrap replicates) based on multiple alignments with ClustalW

The phylogenetic relationships between BvHATs and other HATs from various plant species, such as *Arabidopsis thaliana (At), Solanum lycopersicum (Sl),* and *Oryza sativa (Os)*, were determined using ClustalW and MEGA11 software. According to the phylogenetic tree (Fig. 1), a total of 39 HAT proteins from 4 plant species were clustered into four major groups including GNAT (GCN5, ELP3, and HAT1), MYST, CBP, and TAFII250 family, which were represented by different colors in Fig. 1. However, HPA2 family including 12 tomato HAG proteins (SlHAG7-8, and SlHAG15-25) did not contain any *B. vulgaris* HAT proteins. Aiese Cigliano et al. (2013) reported that the tomato HPA2 family includes 23 HAGs. They found the HPA2-like proteins that were the largest group of HAGs in *Arabidopsis* and tomato. In the present work, three protein families from GNAT superfamily (GCN5, HAT1, ELP3) consisted of BvHAG2 (orange), BvHAG3 (red), and BvHAG4 (blue), respectively. BvHAG1 was closely related to AtHAG4, and AtHAG5 in MYST family. The BvHAC1 (green) involved in CBP class was related to SIHAC3, SIHAC4, and AtHAC2. Another protein at the CBP class, the BvHAC2 (green) was found at the same clade with SIHAC1, and SIHAC2 proteins (Fig. 1). BvHAF1 belonging to TAFII250 family was found at the same clade with SlHAF1.

### Conserved motifs and structure of *BvHAT* genes

**Fig. 2:**
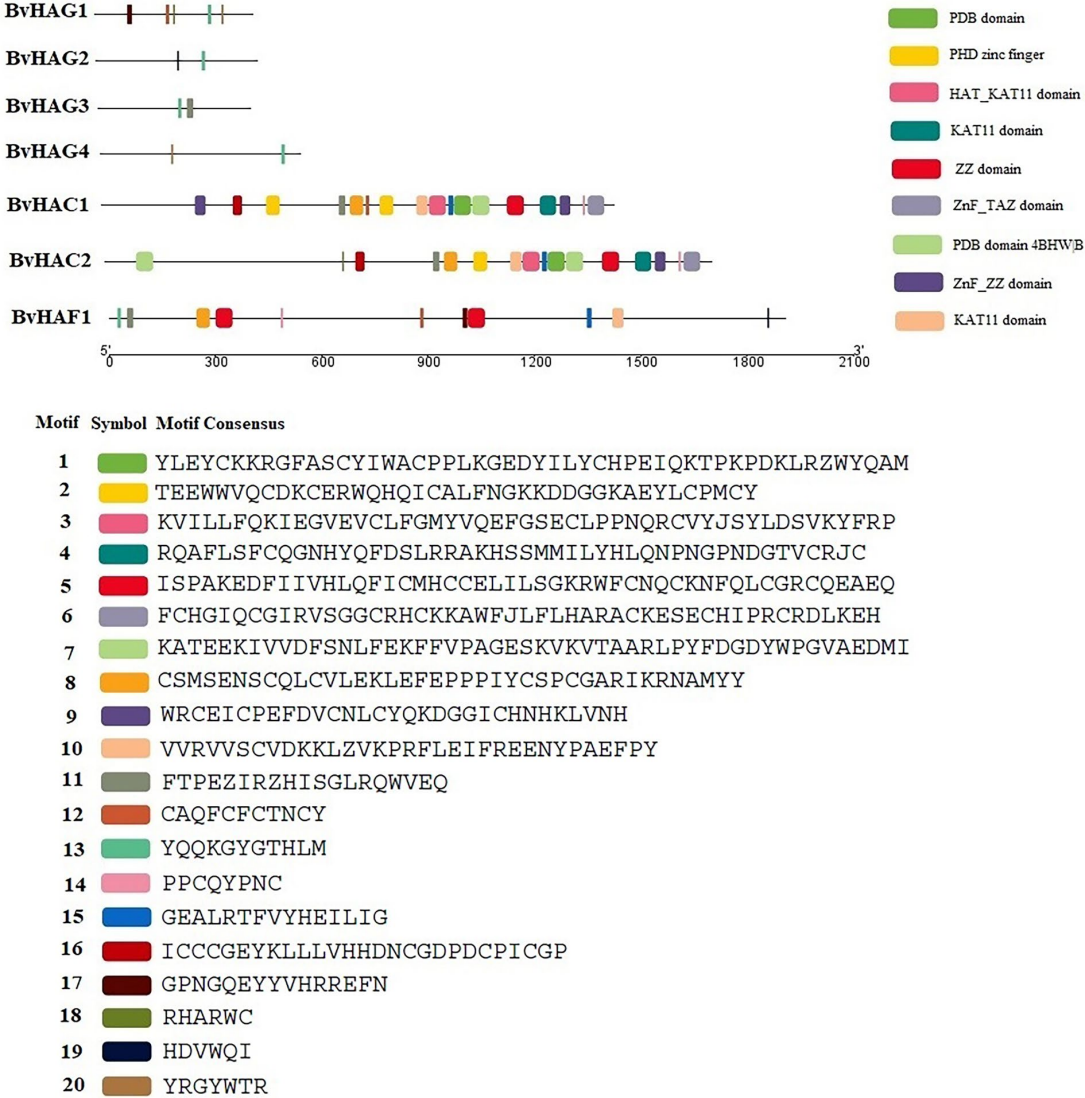
Motif analysis of the HAT proteins in *B. vulgaris*. The MEME online tool and TBtools were used to analyze and draw the conserved domains. Different motifs are indicated by different colors and numbers

MEME analysis was performed to examine the structural diversity of HAT proteins and predict the conserved motifs (Bailey and Elkan 1994). A total of 20 motifs were determined in 7 sugar beet HAT proteins, and they were highlighted with different colors (Fig. 2). Their amino acid lengths varied ranging from 6 to 50. They showed variation in the numbers and types of conserved motifs. The motif 4 (KAT11 domain, which participates in H3K56 acetylation) was present in HAC1, and HAC2. All HAT proteins except for HAC1, and HAC2 were found to contain motif 13. Two CBP members, BvHAC1 and BvHAC2 existed similar motifs, suggesting that these proteins may have similar functions. This finding is highly consistent with the phylogenetic tree. The motifs 1, 2, 3, 6, 7, and 9 were present only in BvHAC1 and BvHAC2 protein sequences. The motif 6 is ZnF_TAZ domain that is zinc-containing domain found in the CBP and the P300. PHD zinc finger, the plant homeodomain (PHD) finger (motif 2) existed in HAC proteins. In addition, the CBP proteins had the maximum numbers of motifs, consistent with *Triticum aestivum* HAC proteins (Gao et al. 2021). There were 17 and 16 motifs in the same order, respectively. Four motifs including 5, 8, 10, and 14 were present in HAC1, HAC2 and HAF1. Motif 5 is ZZ domain (zinc finger), which includes 49 amino acids in length. The minimum numbers of motifs were found in BvHAG2, BvHAG3, and BvHAG4 proteins. The conserved domains of BvHAG1, BvHAG2, BvHAG3, and BvHAG4 were reported to possess the shortest amino acid lengths as compared to BvHAC1, BvHAC2 and BvHAF1.

**Fig. 3:**
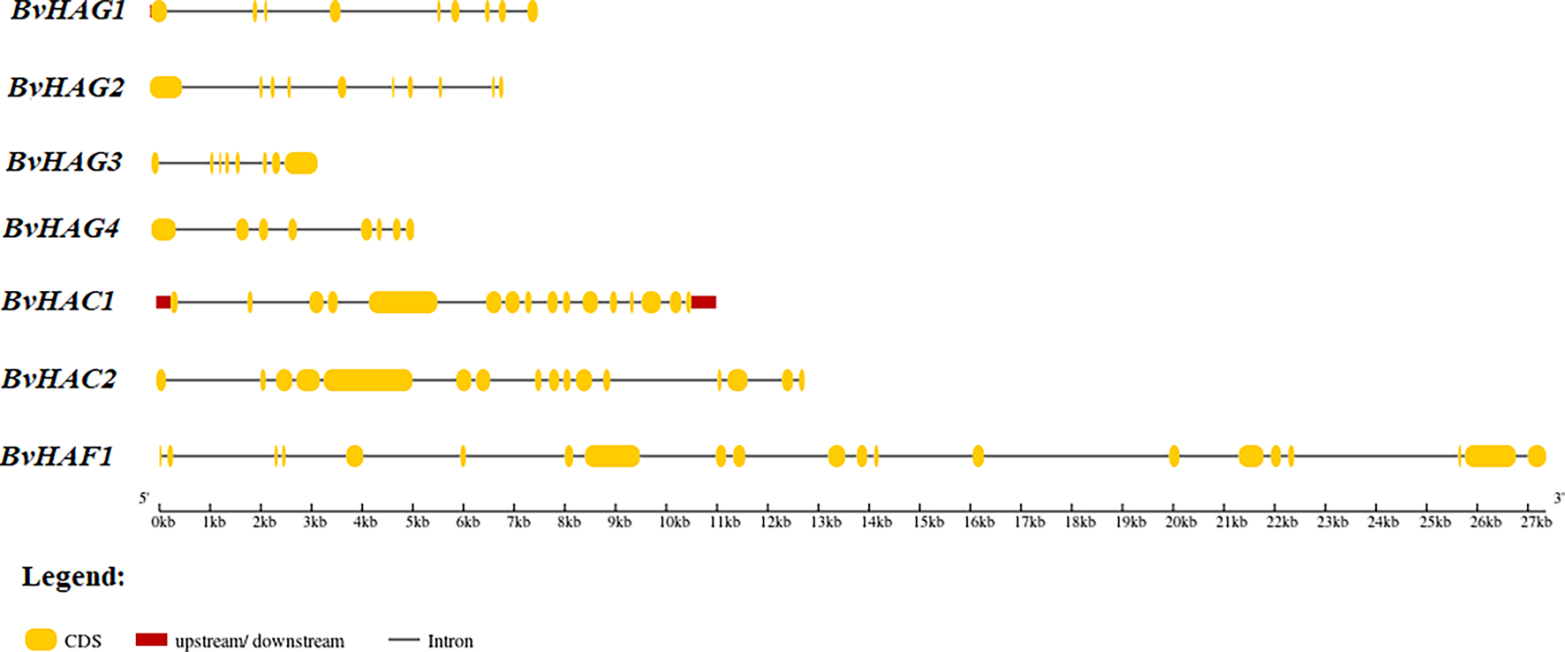
Gene structure analyses of the *BvHATs* performed in GSDS 2.0 tool. Exons and introns are indicated by yellow boxes, and black lines, respectively. Kb: kilo bases

The numbers, the lengths, and the organization of introns and exons impact gene expression and functions (Xu et al. 2012). Comparing the gene structures provides insights into the evolution of gene families. The exon–intron structures of seven *BvHATs* were examined and visualized according to CDS and genomic sequences of each *B. vulgaris HAT* gene. The results are indicated in Fig. 3. The number of introns ranged from 8 to 20, and the exons ranged from 8 to 21. *BvHAG3* and *BvHAG4* belonging to HAT1 and ELP3 subfamily had both 7 introns and 8 exons, and their gene structures appeared similar to each other. *BvHAC1* and *BvHAC2* genes also have highly similar gene structures, and they contain 15 introns, and 16 exons. They were also clustered together at the phylogenetic tree. The *BvHAF1* contains the highest numbers of introns (20) and exons (21) among *B. vulgaris HAT* genes.

### Chromosomal locations and Ka/Ks calculation

**Fig. 4:**
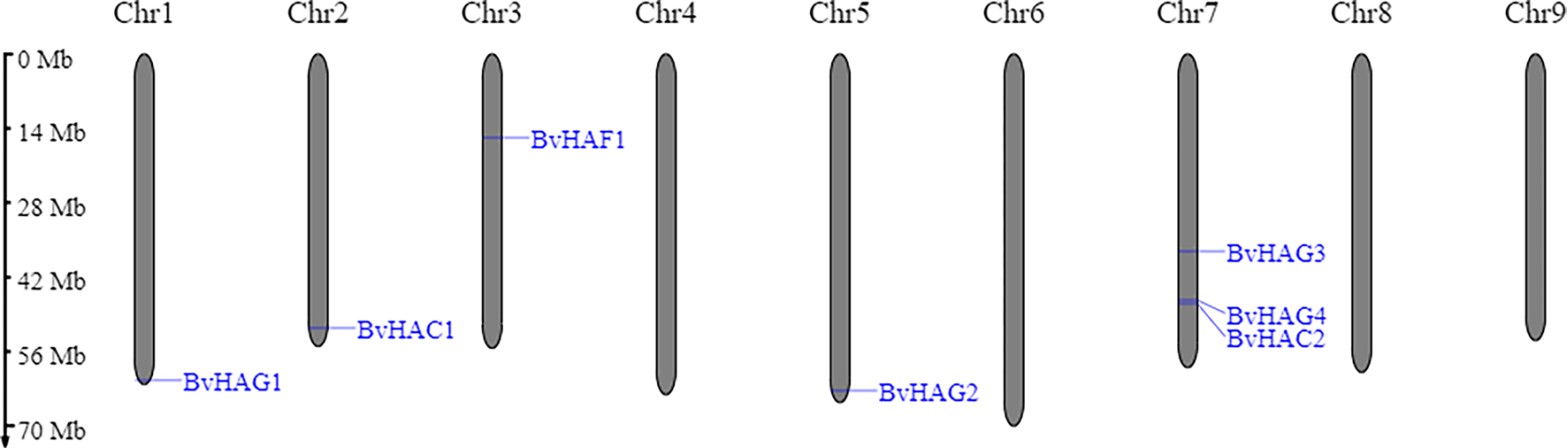
Chromosomal positions of seven *HAT* genes in sugar beet genome generated in MG2C tool. The chromosome number is shown at the top of each chromosome. The genome scale in megabases (Mb) is given on the left

To investigate the chromosomal distribution of the *BvHAT* genes, they were mapped on the chromosomes using the MG2C v2.1 tool. Chromosome information was obtained from the sugar beet genomic database. The sugar beet *HAT* gene family was found to be dispersed on chromosomes 1, 2, 3, 5, and 7 (Fig. 4). The *HAG3, HAG4* and *HAC2* are located on chromosome 7, while the *HAC1* and *HAF1* were found on chromosomes 2, and 3, respectively. Two genes (*HAG1*, and *HAG2*) belong to MYST and GCN5 class were found on chromosomes 1 and 5. No *HAT* genes were found on chromosomes 4, 6, 8, and 9.

To further investigate the evolutionary selective pressure on *HAT* genes, we calculated Ka/Ks ratio, which was lower than one for six *BvHAT* genes. This finding exhibited that the paralogous gene pairs developed under purifying/negative selection (Table 3). The Ka/Ks ratios were calculated 0.8854 for *HAF1-HAG4*, 0.8543 for *HAC2-HAG2*, and 0.9043 for *HAC1-HAG1* gene pairs. The divergence time for 3 paralogous gene pairs was found to be as 3.82, 2.91, and 2.63 million years ago (Mya), respectively. Purifying selection removes mutations and helps preserve gene functions within the population.

**Table 3:** Analysis of synonymous (Ka) and non-synonymous (Ks) substitution rates in *BvHAT* paralog genes, and their divergence time

Paralogous gene pairs	Ka	Ks	Ka/Ks	Time (Mya)
*BvHAF1-BvHAG4*	0.4733	0.5346	0.8854	3.82
*BvHAC2-BvHAG2*	0.3481	0.4075	0.8543	2.91
*BvHAC1-BvHAG1*	0.3326	0.3678	0.9043	2.63

### Sequence similarity with Circos

**Fig. 5:**
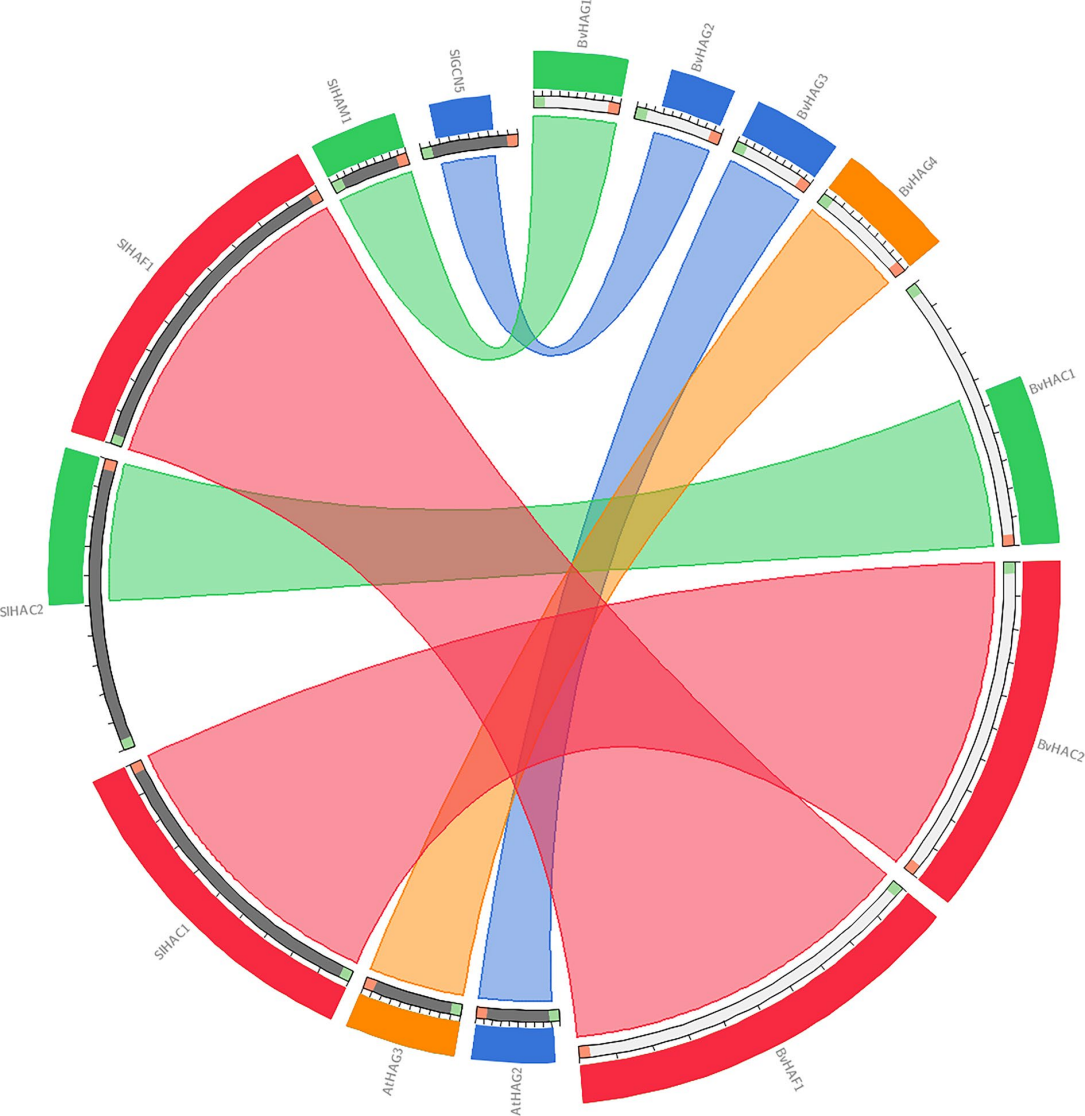
Syntenic relationships of HATs among sugar beet and rice, tomato, *Arabidopsis*. Red, orange, blue, and green lines which connect two proteins indicate synteny between sugar beet and other plants

Synteny circos figure indicated the sequence similarity between sugar beet and other plants. The colors in Fig. 5 represented the level of evolutionary conservation among *HAT* genes. The tool used ‘score/max’ ratio coloring with blue ≤ 0.25, green ≤ 0.50, orange ≤ 0.75, and red > 0.75 (Darzentas 2010). The ribbons were colored by bitscore. It was concluded that five of the *B. vulgaris HAT* genes and *S. lycopersicum HAT* genes had similar evolutionary origin, they had high amino acid sequence similarities. The highest sequence similarity greater than 75% was observed between SlHAF1-BvHAF1, and SlHAC1-BvHAC2 proteins. Moderate similarity lower than 50% was seen in SIHAC2-BvHAC1, and SIHAM1-BvHAG1. BvHAG1 originated from *S. lycopersicum* HAM1. HAG3 and HAG4 from *B. vulgaris* showed synteny with *Arabidopsis* HAG2, and HAG3 proteins, respectively. The lowest similarities were demonstrated in SIGCN5-BvHAG2, and AtHAG2-BvHAG3 (Fig. 5).

### *Cis-*acting regulatory elements in promoter regions of *BvHAT* genes

**Fig. 6:**
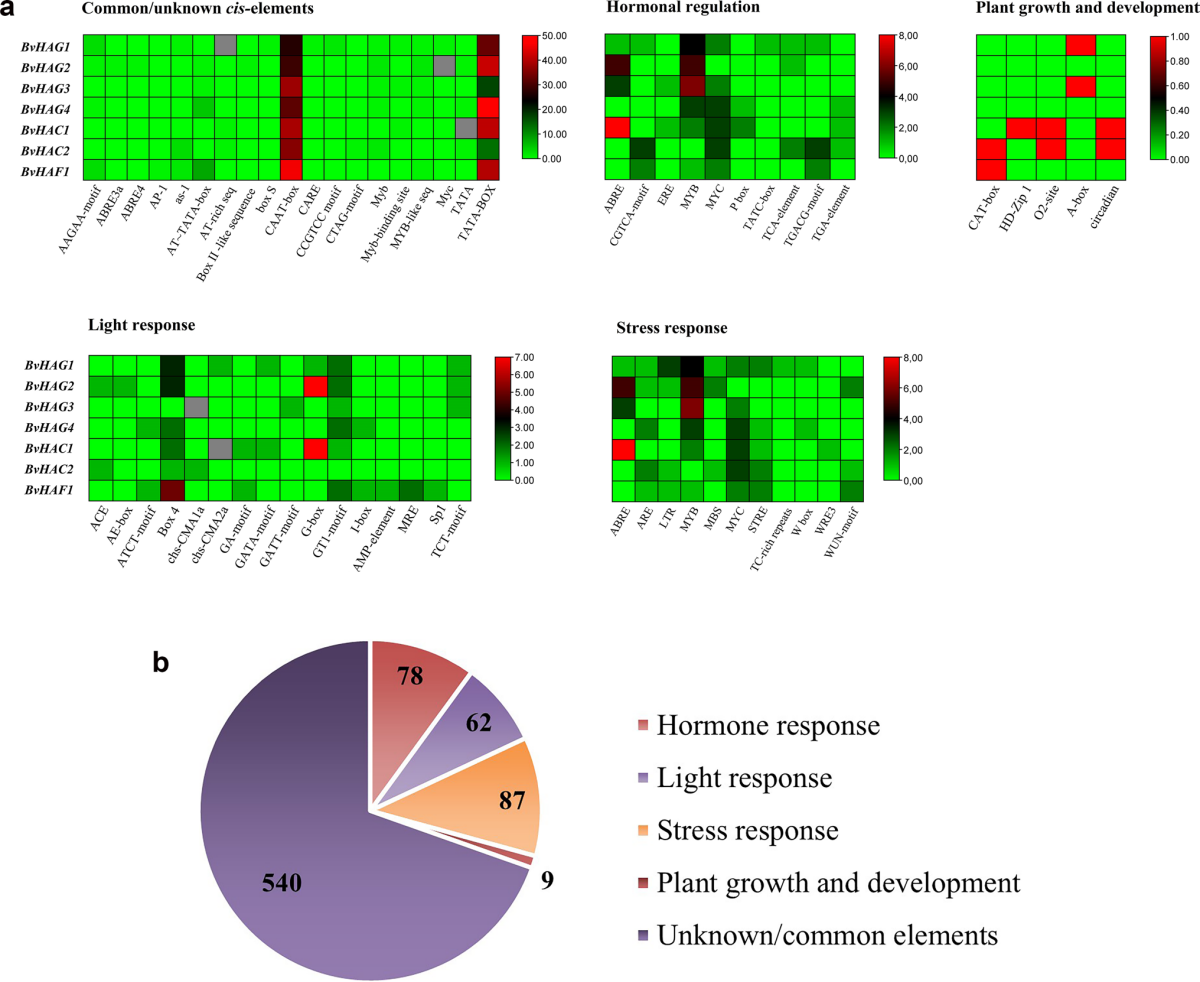
**A** Functional classification of *cis*-acting elements through heatmaps, which were constructed using by TBtools. The numbers of *cis-*acting elements were visualized by different colors as shown in heatmap color scale. Colors from green to red represent low to high amounts of *cis*-elements. Red color indicates maximum numbers. **B** Total numbers and functions of *cis*-acting regulatory elements related to hormone, light, stress response, plant growth, and development (endosperm and meristem expression, differentiation of the palisade mesophyll cells, zein metabolism, and alpha-amylase) which were predicted by PlantCARE software

To predict functional characteristics of *BvHAT* genes, *cis-*elements in promoters were analyzed by searching 1500 bp upstream region of transcriptional activation site. PlantCARE results exhibited 58 types of *cis-*acting elements, which were classified into five different groups: common or unknown promoter elements (19), hormone response (10), light response (16), stress response (11) and plant growth and development (5) (Fig. 6A, B). A total of 776 *cis-*elements were obtained: **light response** (*ACE, AE-box, ATCT-motif, Box 4, chs-CMA1a, chs-CMA2a, GA-motif, GATA-motif, G-box, GATT-motif, GT1-motif, I-box, LAMP-element, MRE, Sp1, TCT-motif*), **abscisic acid** (*ABRE, MYC, MYB*), **auxin** (*TGA-element*), **gibberellin/MeJA** (*TGACG-motif, TATC-box, CGTCA-motif*), **salicylic acid** (*TCA-element*), **ethylene** (*ERE*), **low temperature** (*LTR*), **high temperature** (*WRE3*), **drought** (*ABRE, MYB, MYC, MBS*) and **stress response elements** (*TC-rich repeats, STRE*), **meristem expression** (*CAT box*), **endosperm expression** (*HD-Zip1*), **differentiation of the palisade mesophyll cells** (*O2 site*), **zein metabolism** (*A box*), and **circadian control** (*circadian*). The first largest group was common or unknown *cis-*elements with 540 members, while the second largest group was stress response-specific elements. *BvHATs* were found to contain higher levels of *ABRE* (17), *MYB* (21), *MYC* (15), and *G-box* (15) related to hormonal regulation, stress response and light response. Ethylene-responsive element, *ERE* was predicted to be present in *BvHAG3*, and *BvHAC1* genes. There were no *ABA-responsive elements (ABREs)* in three *HAT* genes, *BvHAG4*, *BvHAC2*, and *BvHAF1*. Instead, *ABRE* is found in the promoters of *BvHAG1, BvHAG2, BvHAG3,* and *BvHAC1*. All *HAT* genes except for *BvHAG2* included *MYC* that was previously reported to be involved in chilling response (Zhang et al. 2016). The highest number of *cis-*elements was observed in the promoter regions of *BvHAF1* and *BvHAC1* genes. In addition to *ABRE, STRE* and *MYC*, the *low temperature-responsive element (LTR)*, *MYB binding site (MBS), TC-rich repeats,* and *WRE3* were found to be correlated with abiotic stress, such as low temperature, drought, and heat. *TC-rich repeats* existed only in the *BvHAG1* gene. The *TGA-element* associated with auxin responsiveness was present only in *BvHAG4, BvHAC1,* and *BvHAC2*. Two types of *cis-*elements involved in MeJA response (*TGACG-motif, CGTCA-motif*) existed in *BvHAG1, BvHAC2,* and *BvHAF1* promoters. Except for the *BvHAG3, BvHAG4*, *BvHAC2* and *BvHAF1* genes, the remaining 3 *HAT* genes contained 15 *G-box*, which is associated with light response. Two elements called *HD-Zip1,* and *O2 site* which are correlated with endosperm expression and differentiation of the palisade mesophyll cells, were found only in the *BvHAC1* promoter. Furthermore, there were two *CAT-box* elements in *BvHAC2* and *BvHAF1* that are associated with meristem expression. *A box* was present in the *BvHAG1* and *BvHAG3* promoters. An alpha-amylase-specific *cis-*element, *circadian* existed in two CBP class genes, *BvHAC1* and *BvHAC2*.

Consequently, PlantCARE tool predicted different *cis*-elements related to promoter regions (540), hormone response (78), light response (62), stress response (87), plant growth and development (9) according to functional classification (Fig. 6B).

### Protein 3D structure

**Fig. 7:**
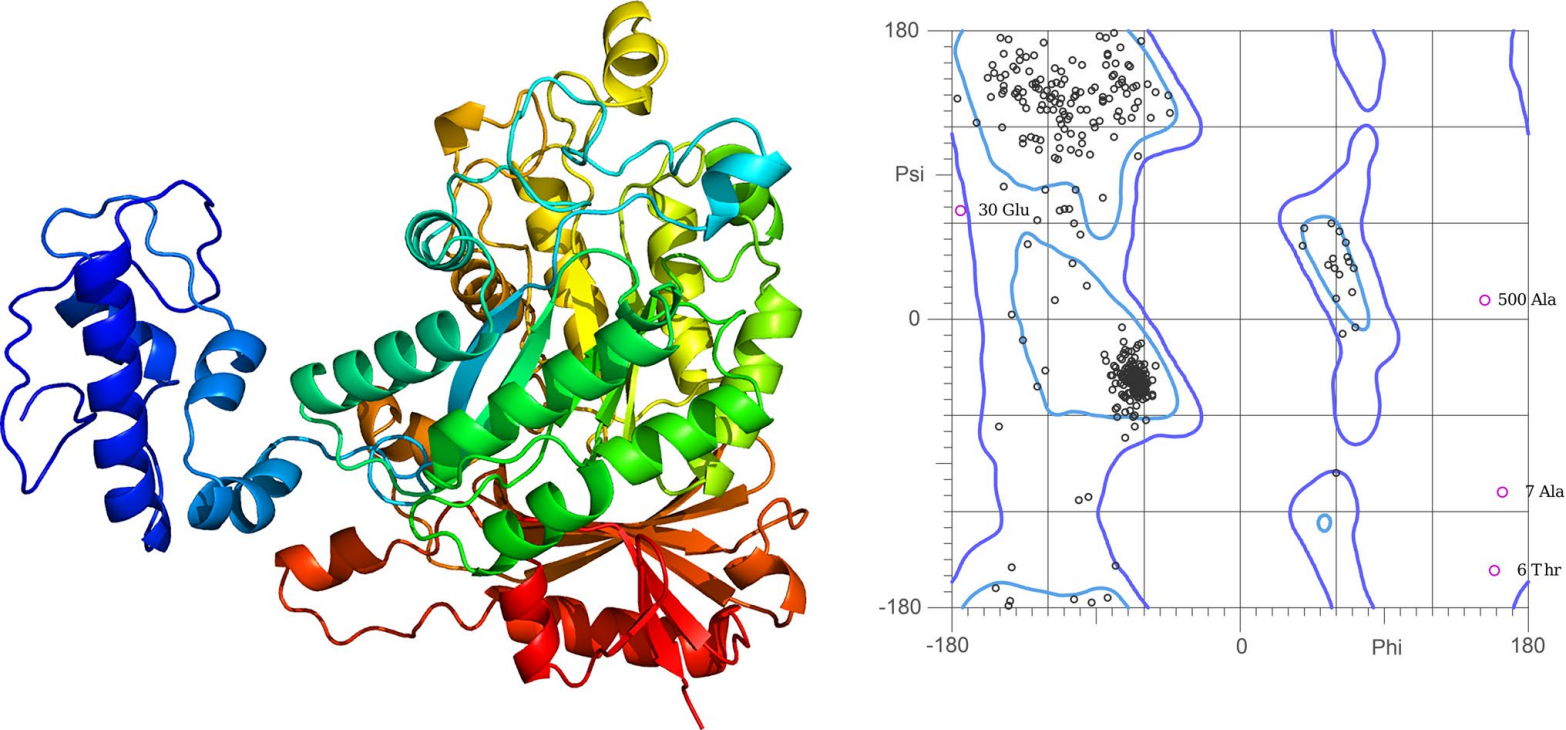
3D model and Ramachandran plot of BvHAG4 protein. The protein model was constructed in the Phyre2 web portal

Phyre2 web portal was used to construct *B. vulgaris* HAT protein structures. 3D models of BvHATs generated with > 90% confidence at 46–95% residue coverage. The highest coverage was observed in BvHAG4 (95%), BvHAG1 (92%), BvHAG3 (74%), BvHAC1 (75%), BvHAG2 (52%), BvHAF1 (47%) and BvHAC2 (46%), respectively. BvHAGs had a higher coverage rate than other BvHACs and BvHAF1. The 3D protein structure and Ramachandran plot analysis of the BvHAG4 protein with the highest coverage and confidence are shown in Fig. 7. In terms of coverage and constructed 3D models, BvHAG1 and BvHAG4 proteins had the highest coverage and confidence, but other BvHATs had lower results, especially BvHAG2, BvHAC2 and BvHAF1 proteins. Most of the amino acid sequence (65%) of BvHAF1 has not been modeled. Secondary structure analyses revealed that the α-helices are the major secondary structure of BvHATs with the range of 32.38% (BvHAC2) – 43.64% (BvHAG4), while β-strands are distributed in the range of 10.99% (BvHAC2) – 17.90% (BvHAG1).

Templates used to construct 3D structural homology included the acetyltransferase domain of human HIV-1 Tat interacting protein (2OU2), human HBO1 in complex (5GK9), human MYST histone acetyltransferase (2GIV), Elongator catalytic subcomplex Elp123 lobe (6QK7), yeast Elp123 in complex (8ASW) and so on. The templates contained transferase domains, such as GNAT, N-acetyl transferase like, GCN5-related N-acetyl transferase, MYST family zinc finger, winged helix DNA-binding, HAT1 N-terminal domain, IKI3 protein domain, and some other transferase domains.

### Expression patterns of *B. vulgaris HAT* genes under salt stress

**Fig. 8:**
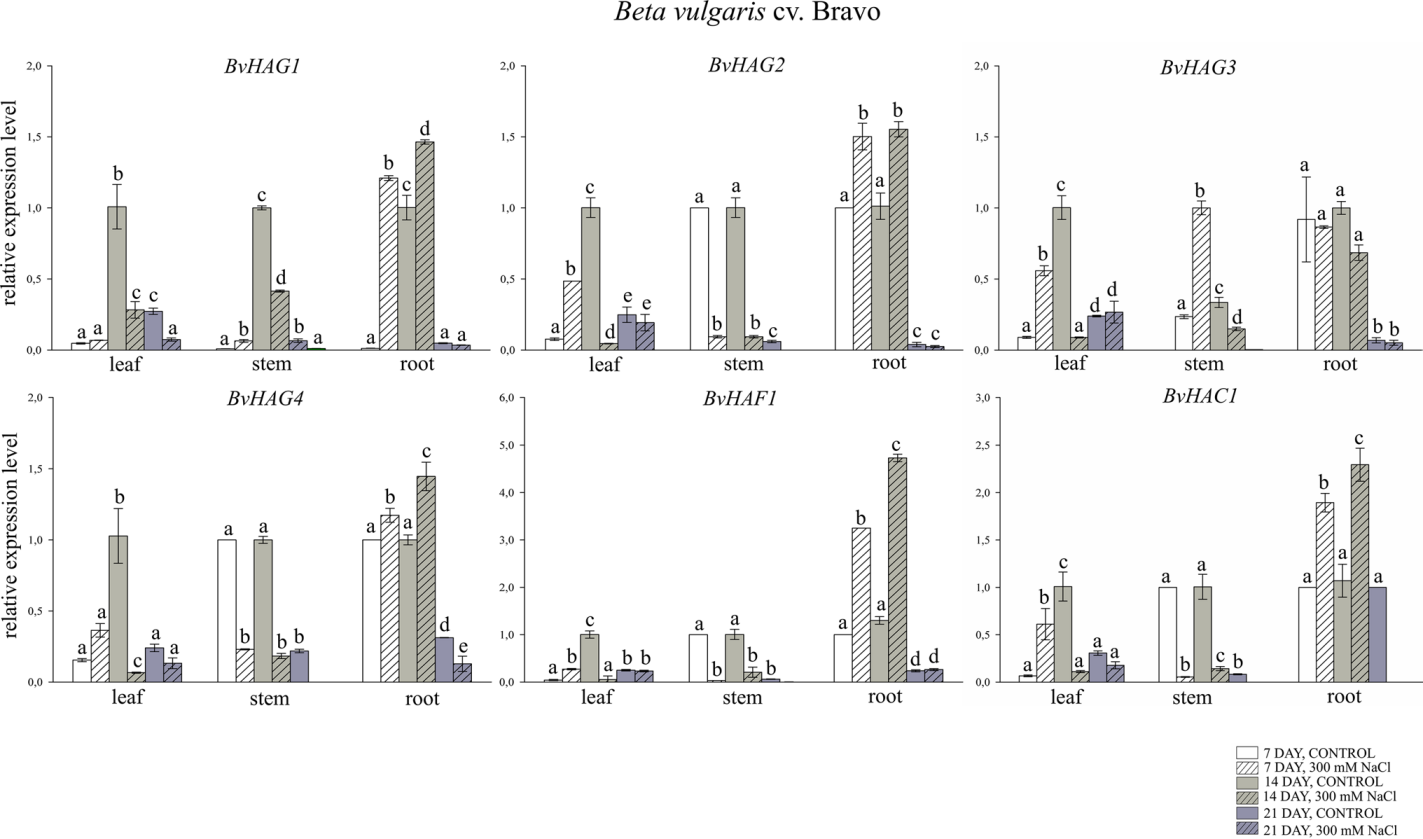
Expression profiles of *BvHAG1*, *BvHAG2*, *BvHAG3*, *BvHAG4*, *BvHAF1* and *BvHAC1* genes determined in leaves, stems, and roots of *Beta vulgaris* cv. Bravo on the 7th, 14th, and 21st day of salt stress treatment. 7th day, control − white bars, 7th day, 300 mM NaCl – white bars with cross stripes, 14th day, control – gray bars, 14th day, 300 mM NaCl – gray bars with cross stripes, 21st day, control – dark gray bars, 21st day, 300 mM NaCl – dark gray bars with cross stripes. Different letters denote significant differences at p < 0.05 (ANOVA followed by Tukey’s test). “Whiskers” indicates standard deviation

**Fig. 9:**
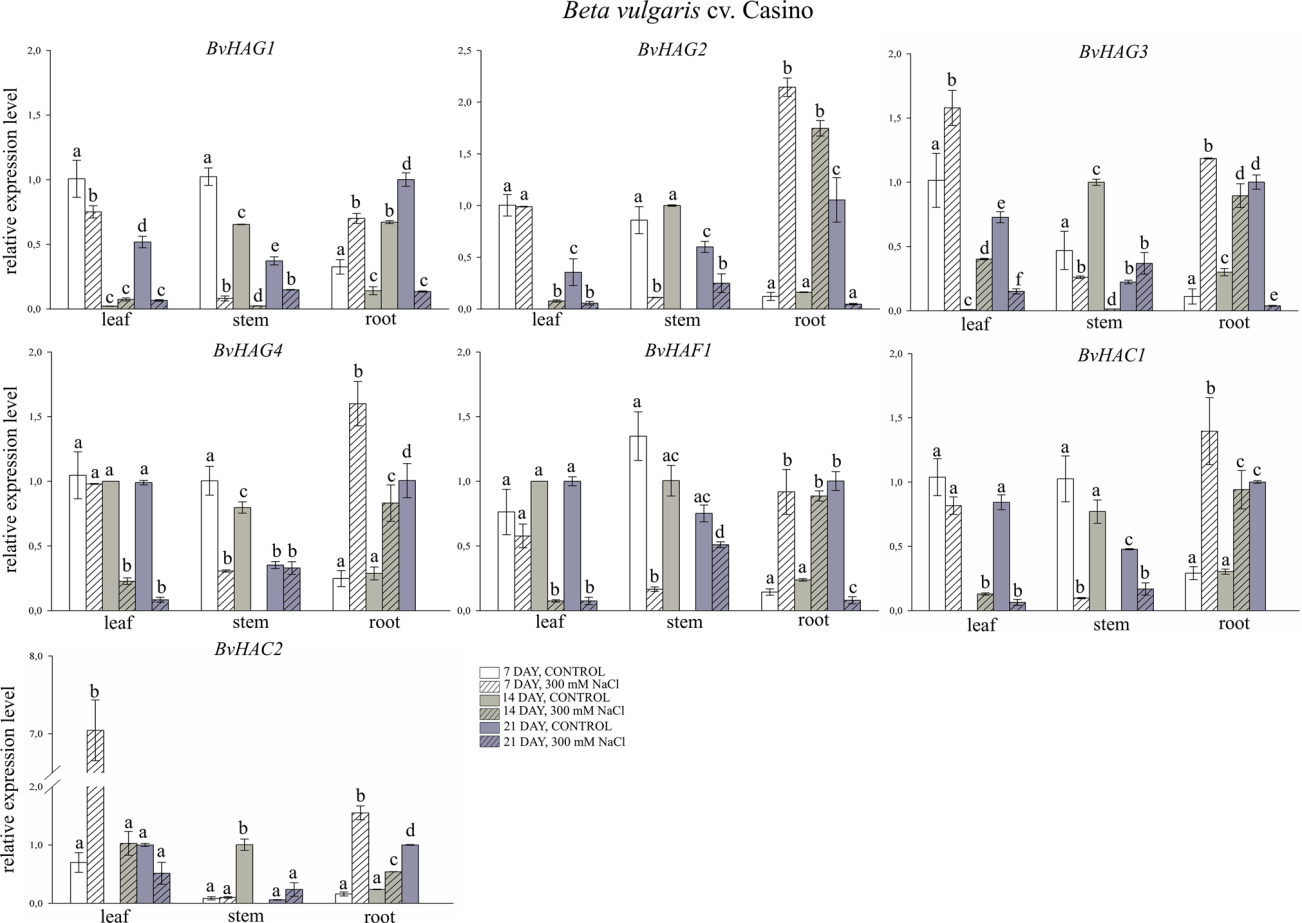
Expression profiles of *BvHAG1*, *BvHAG2*, *BvHAG3*, *BvHAG4*, *BvHAF1*, *BvHAC1* and *BvHAC2* genes determined in leaves, stems, and roots of *Beta vulgaris* cv. Casino on the 7th, 14th and 21st day of salt stress treatment. 7th day, control – white bars, 7th day, 300 mM NaCl –white bars with cross stripes, 14th day, control – gray bars, 14th day, 300 mM NaCl –gray bars with cross stripes, 21st day, control − dark gray bars, 21st day, 300 mM NaCl – dark gray bars with cross stripes. Different letters denote significant differences at *p* < 0.05 (ANOVA followed by Tukey’s test). “Whiskers” indicates standard deviation

Transcript levels were determined in three tissues, i.e., leaves, stems and roots, of two genotypes of beet *B. vulgaris* subsp. *vulgaris* L. cv. Bravo and *B. vulgaris* subsp. *vulgaris* L. cv. Casino, grown under control conditions or treated with 300 mM NaCl (Figs. 8 and 9). In roots of *B. vulgaris* cv. Bravo, a significant increase was observed in the expression levels of genes encoding *BvHAG1* (1.2-fold; 1.4-fold increase, respectively), *BvHAG2* (1.5-fold; 1.6-fold), *BvHAG4* (1.2-fold; 1.5-fold increase, respectively), *BvHAF1* (3.2-fold; 4.7-fold) and *BvHAC1* (1.8-fold; 2.3-fold) on the 7th and 14th days of salt stress exposure, as compared to control (Fig. 8). Importantly, the *BvHAC2* gene in *B. vulgaris* cv. Bravo was not expressed in any experimental variant tested (Fig. 8). In the remaining experimental variants, decreases in the expression of the tested genes were observed, although these decreases were in most cases significantly greater in stress-treated samples compared to the control. The exception was decreases in the expression of the *BvHAG2* and *BvHAG3* genes in leaves, which in samples treated with salt stress were significantly lower compared to the control. In the roots of *B. vulgaris* cv. Casino, a significant increase in *BvHAG2* expression was observed (2.1-fold; 1.7-fold), compared to the control after 7 and 14 days of salinity. A significant increase in the transcription of genes encoding *BvHAG3* (1.2-fold), *BvHAG4* (1.6-fold), *BvHAC1* (1.3-fold) and *BvHAC2* (1.5-fold) in this sugar beet genotype was demonstrated in roots treated with salt stress, compared to the control, on the 7th day of the experiment. Moreover, a significant seven-fold increase in the expression level of the *BvHAC2* gene and a 1.6-fold increase in the expression level of the *BvHAG3* gene, compared to the control, occurred in the leaves of *B. vulgaris* cv. Casino, treated with salt stress on day 7. In the remaining experimental variants, declines in the transcript levels were seen in comparison to control samples (Fig. 9). However, in the roots of the tested genotype on the 7th and 14th day of salt stress treatment, the decreases in the expression levels of the *BvHAG1* and *BvHAF1* genes were significantly lower in the treated samples compared to the controls. A similar expression profile was observed on day 14th of the experiment for *BvHAG3, BvHAG4, BvHAC1* and *BvHAC2* transcripts (Fig. 9).

## Discussion

Epigenetic modifications, such as histone acetylation, phosphorylation, DNA methylation, etc. regulate gene expression under different environmental conditions (Yuan et al. 2013). In eukaryotes, HATs add the acetyl group to the core histone tails and make DNA accessible for transcription. HAT family members play regulatory roles in the form of complexes in cellular processes, such as gene transcription/silencing, cell cycle regulation, DNA replication and repair, and chromosome assembly (Mai et al. 2009). They also play an important role in plant growth, development, fruit ripening, and response to biotic and abiotic stress factors (Chen and Tian 2007; Cai et al. 2022). Although salinity and drought bring about yield loss in sugar beet (Clarke et al. 1993), number of reports on the relationship between abiotic stress tolerance and epigenetic mechanisms is limited. To understand the underlying mechanisms of stress tolerance in sugar beet, further research including bioinformatics approach or experimental works needs to be performed. In this work, an in silico analysis of *B. vulgaris HAT* genes was performed using bioinformatics tools/databases and their expression patterns were determined in different tissues, such as stems, roots, and leaves of salt-resistant (*B. vulgaris* cv. Casino) and -sensitive (*B. vulgaris* cv. Bravo) sugar beet genotypes under salt stress. Seven HATs were identified in *B. vulgaris* and they were phylogenetically divided into 4 major HAT groups: GNAT, MYST, CBP and TAFII250, parallel with previous reports (Aquea et al. 2010; Pandey et al. 2002; Aiese Cigliano et al. 2013; Shu et al. 2021). The largest family, GNAT was found to have three sugar beet HAT proteins, including HAG2 (GCN5), HAG3 (HAT1), and HAG4 (ELP3). Sugar beet has fewer HATs than *Arabidopsis* (Pandey et al. 2002), tomato (Aiese Cigliano et al. 2013; Hawar et al. 2021), pepper (Cai et al. 2022), citrus (Shu et al. 2021), foxtail millet (Xing et al. 2022), and wheat (Gao et al. 2021). However, similar to sugar beet, some plant species also had lower numbers of HATs, such as grapevine (Aquea et al. 2010), litchi (Peng et al. 2017), and rice (Liu et al. 2012). It is important to note that the variation in numbers of HATs in different plants may be related to species-specific diversification or gene duplication during evolution. The homolog genes of *Arabidopsis* HATs belonging to GCN5, ELP3, HAT1, MYST, CBP, TAFII250 subfamilies were detected in sugar beet. Consistently, in wheat genome, all *Arabidopsis* HATs correspond to homologous genes (Li et al. 2022). Additionally, 71% of BvHATs showed amino acid similarities with *S. lycopersicum* HAT proteins. No HAT proteins from rice indicated similarities with sugar beet proteins. Taken together, sugar beet HATs showed higher similarity with tomato and *Arabidopsis* proteins. In a previous report, in two monocot plant species, foxtail millet (*Setaria italica* L.) and rice, *HAT* genes were found to be genetically similar, and some *SiHAT* genes were likely to be evolved from *Arabidopsis* and rice (Xing et al. 2022). Based on Ka/Ks calculation, *BvHAT* gene pairs underwent purified selection, consistently with *S. italica* (Xing et al. 2022), and *T. aestivum HAT* genes (Gao et al. 2021). The Ka/Ks ratios in *BvHAF1-BvHAG4* and *BvHAC-BvHAG2* gene pairs were less than 0.9, indicating that these genes may be subjected to relaxed purification selection (Dong et al. 2019).

According to MEME data, each HAT subfamily member had specific domains, which were consistent with phylogenetic analysis. The structures of different HAT subfamily members exhibited variation in amino acid lengths and type of domains. For instance, there are two members (HAC1 and HAC2) of CBP family. CBP members, HAC1 and HAC2 contained PHD domain (motif 2), HAT KAT11 superfamily (motif 3), ZnF_TAZ domain (motif 6), and ZnF_ZZ domain (motif 9). Two HAC proteins containing identical motifs may show functional similarities. The GNAT family proteins (HAG2, HAG3, and HAG4) contained acetyltransf_1 domain. The protein sequences used to predict the 3D structures were taken from the genome of *B. vulgaris* L. subsp. *vulgaris* (EL10.2_2) in the Phytozome database v13. Predicting the 3D structure of proteins is critical to understand their biological functions at the molecular level. 3D prediction bridges the gap between sequence and structure and contributes to the annotation of protein–protein interactions (Liu 2017). The homology modeling approach we used relies on experimentally generated 3D proteins for 3D structure prediction (Kelley et al. 2015). The 3D protein models used to construct the 3D structures of the BvHATs were from different organisms and included important HAT domains from MYST (Thomas et al. 2008) and GCN5 (Clements et al. 1999) families. Due to the conserved structure of HAT domains, the 3D models are built using proteins from a wide range of organisms. Based on the generated 3D structures and Ramachandran plot analysis, it was observed that the 3D structures generated using these annotated protein sequences, except for BvHAG1 and BvHAG4, were not generated efficiently. Thus, for the less covered BvHATs, it can be suggested that more sequence curation is needed.

Two subcellular localization prediction tools were used to determine the possible location of HATs in sugar beet. Similar to AtHAF1, AtHAF2, OsHAF701, and ZmHAF101 (Liu et al. 2012), BvHAF1 was assumed to be localized in nucleus. WOLF PSORT predicted nuclear localizations of HAC1, HAC2, and HAF1 with high frequencies (11–14), while HAG2 localization was predicted to be present in chloroplast with high frequency (12). Cello-life exhibited that the HAG1, HAG2, HAG4, HAC1, HAC2, and HAF1 proteins were found in nucleus. Cello-life predictions were consistent with WOLF PSORT results for 57% of sugar beet HAT proteins (HAG4, HAC1, HAC2, and HAF1), while HAG3 was in both the nucleus and cytoplasm. These findings suggested that most HAT proteins in *B. vulgaris* might have nuclear localizations, which is consistent with the HAT proteins in different plant species, such as *T. aestivum* (Li et al. 2022), *S. lycopersicum* (Hawar et al. 2021), and *O. sativa* (Liu et al. 2012). The cytosolic localization of OsHAC701, OsHAG702, and OsHAG704 in *O. sativa*, and lysine acetylation of distinct proteins outside of the nucleus in *Arabidopsis* suggested an important catalytic role of HATs other than histone acetylation (Liu et al. 2012; Wu et al. 2011). Moreover, in *Arabidopsis* and *O. sativa*, lysine-acetylated (LysAc) proteins were found in different cellular compartments, including chloroplast, nucleus, plasma membrane, and so on (Wu et al. 2011).

*Cis-*acting elements in promoters are transcriptional gene regulatory units, and they regulate various biological processes, such as hormonal response, stress response, light response, and development (Schmitz et al. 2022). Different *cis-*elements have specific functions in gene transcription in plants. Here, a total of 718 *cis-*elements have been predicted to be included in the promoter regions of *B. vulgaris HAT* genes that were classified into 4 functional groups. Similar to our findings, several *cis-*elements related to hormone response, light responsiveness, anaerobic induction, and stress response were predicted in the promoters of *HAT* genes in different plant species, such as *Citrus sinensis* (Shu et al. 2021), *Triticum aestivum* (Gao et al. 2021; Li et al. 2022), and *Setaria italica* (Xing et al. 2022). According to PlantCARE data, identical *cis-*elements related to stress responsiveness (LTR, ABRE, MBS, WUN-motif, MBS), and hormone response elements associated with ABA, gibberellin, jasmonate, salicylic acid, and auxin were found in both histone acetyltransferase- and deacetylase-encoding genes (*HATs* and *HDACs*) in sugar beet (Yu et al. 2023). It has been found 87 *cis-*regulatory elements involved in stress response of *B. vulgaris HATs*. Among them, the highest numbers of *cis-*elements were present in *MYB* elements, suggesting the importance of sugar beet *HATs* in abiotic stress response. Recently, promoter analysis showed that *Triticum aestivum* genes, *TaHAG2, TaHAG3* and *TaHAC2* contained a great number of stress-related *cis-*acting elements, such as *STRE* and *ABRE* (Li et al. 2022). In our study, a total of 8 *STREs* were found in *HAG1* (2), *HAG4* (1), *HAC1* (1), *HAC2* (2), and *HAF1* (2) genes. Instead of *STRE*, two genes, *HAG2* and *HAG3* had abscisic acid-responsive elements (ABREs). Four genes, *HAG1* (1), *HAG2* (5), *HAG3* (3) and *HAC1* (8) contained totally 17 *ABREs*, which are involved in ABA-regulated gene expression (Giraudat et al. 1994), suggesting that the regulation of salt stress might be associated with the ABA signaling pathway. A total of 6 low temperature response elements (LTR) were present in *HAG1, HAG2, HAC2,* and *HAF1* promoters. GCN5-type *BvHAG2* gene contained *LTR* element, suggesting that this gene may be responsible for cold response in *B. vulgaris*. Similarly, *Arabidopsis* and *O. sativa HAT* genes were found to be associated with cold stress response. For instance, cold treatment repressed the mRNA abundances of *OsHAC701, OsHAC703, OsHAC704,* and *OsHAG703* genes in *O. sativa* (Liu et al. 2012), while *Arabidopsis* GCN5 was physically interacted with the cold-induced transcription factor CBF1 (Mao et al. 2006). Furthermore, GCN5 brought about higher drought resistance in *Populus trichocarpa* through acetylation of H3K9 (Li et al. 2018). In addition to hormone-, light- and stress-specific *cis-*elements, *BvHAT* genes included the core promoter elements, such as *TATA box* (233), and *CAAT box* (247), which regulate the appropriate initiation of transcription process by RNA polymerase II (Biłas et al. 2016).

Plant hormones and abiotic stresses, such as low temperature, salt, and drought, impact the transcription levels of *HAT* genes (Gao et al. 2021; Shu et al. 2021; Li et al. 2022; Zheng et al. 2019). In the present work, gene expression assays were conducted on two sugar beet genotypes. Consistent with previous reports mentioned above, in this study, we demonstrated that the salt stress caused alterations in the expression of *BvHAC1* gene in the roots of cv. Bravo, while in the *BvHAC1* and *BvHAC2* genes in the roots of cv. Casino. Importantly, the highest transcriptional activity was observed in roots. In cv. Casino, an increase in *HAT* gene expression was also recorded in leaves. Interestingly, the *HAC2* gene transcription was not affected at all in sensitive plants. This may be some basis for higher stress tolerance in stress-resistant cultivar. However, the expression levels were significantly changed even in control samples. This may indicate that the HAT proteins might be required for the growth and development of sugar beet. According to gene transcript levels, it has been suggested that the *HAT* gene expression depends on the sugar beet genotype and the studied tissue. Besides, the transcription patterns within sugar beet organs might show a broad functional role of HATs. Hence, more detailed wet-lab studies are required to understand the relationship between abiotic stress response and *B. vulgaris HAT* genes. Similarly, the grapevine genes *HAG6902, HAG6904* and *HAC6903* were expressed in the roots, leaves, flowers, and fruits, suggesting their possible involvement in development of *Vitis vinifera* plants (Aquea et al. 2010). On the other hand, four genes including *HAG6901, HAG6903, HAF6901,* and *HAC6902* in grapevine were transcribed in an organ-specific manner, suggesting that these *HAT* genes may have gained different functions during grapevine development. However, the *HAG6901* gene was transcribed exclusively in flowers, indicating its participation in the floral organ development (Aquea et al. 2010). In *Triticum aestivum*, six *HAT* genes were induced in different tissues, such as top leaf, middle leaf, bottom leaf, stem, and root under low temperature, and virus infection (Gao et al. 2021). Consistent with our results, transcript levels were higher in roots under low temperature, and virus infection. All *TaHAT* genes indicated moderate transcription abundances in the stems, and higher expression in the bottom and top leaves than the middle leaves. The transcript levels were lower at low temperatures (8 °C) on days 7–10. As the stress duration increased, the transcript levels of *TaHAF, TaHAG1* and *TaHAG2* genes were higher at 20 °C than at 8 °C (Gao et al. 2021). In a recent study, three *Triticum aestivum* varieties with different drought resistance levels showed variations in *HAT* gene expression (Li et al. 2022). The expression levels of *TaHAG2, TaHAG3, TaHAC2* genes were remarkably higher in the drought-resistant variety, BN207 as compared to other varieties, BN64, and ZM16. In drought-stressed *Citrus sinensis*, all *CsHATs* were transcribed in the roots, but their expression levels were different. Drought stress caused a significant increment in the expression of *CsHAT6, 13,* and *14* genes, and a decline in *CsHAT5* and *CsHAT8* expression (Shu et al. 2021). The transcription of CBP member, *CsHAT13* was significantly induced by drought, which is also seen in *HAC1* gene in *B. vulgaris* roots under salt stress. In a very recent study, transgenic *Arabidopsis* plants expressing a *HAC1* gene from *Medicago truncatula (MtHAC1)* displayed an increase in transcript levels of *HAC1* gene after 24 and 72 h of 150 mM NaCl stress, suggesting the involvement of HATs in plant salt stress response. Besides, *Arabidopsis HAC1*^*RNAi*^ lines showed a delay in the response to salt stress, and elevated expression levels of *HAC1* gene at the 48th hour of salinity exposure (Ivanova et al. 2023). Similar to *Citrus HAT14*, a TAFII250 family member, *HAF1* gene in *B. vulgaris* roots was upregulated by salinity stress. Further studies need to be done to reveal how and whether the *BvHAT* genes respond to the different environmental stresses, such as drought, low temperature, and high temperature.

The presented findings offer insight into the potential roles of sugar beet *HAT* genes and their expression profiles in various tissues under salinity stress. Genome-wide analyses together with gene expression assays under salinity stress may allow the plant biologists/breeders to select and functionally characterize the BvHATs responsible for better stress tolerance in sugar beet cultivars. Further studies are required to confirm the functions of the candidate *BvHAT* genes. It is still unknown how and whether the *BvHATs* respond to different environmental factors, such as drought, heat, and cold, and their subcellular localizations. Hormone and light response-specific *cis-*elements were found to be present in the *BvHAT* promoter regions. However, up to date, no experimental findings have yet been reported on how and whether the *HAT* genes of *B. vulgaris* respond to hormone treatments and light conditions. It is also necessary to find out which histone modifier proteins interact with BvHATs.

## Conclusion

HATs are involved in distinct biological processes, such as abiotic/biotic stress response, growth, development, flowering, etc., in plants. Seven histone acetyltransferase-encoding genes *(HATs)* were identified from *B. vulgaris* L. genome and their expression patterns were analyzed under salt stress. Sugar beet HATs were phylogenetically divided into 4 families: GNAT, MYST, CBP, TAFII250. The prediction tools indicated the nuclear localizations of BvHATs. The ratio of Ka/Ks (non-synonymous/synonymous substitution) demonstrated purifying selection on *BvHAT* genes during evolutionary history. Prediction of *cis-*elements showed potential roles of *BvHAT* genes in abiotic stress response, light responsiveness and hormone regulation. The *BvHAT* genes were differentially transcribed in leaves, stems, and roots under control and 300 mM NaCl stress in *B. vulgaris* salt-resistant (Casino) and -sensitive (Bravo) cultivars. Higher expression levels were observed in roots, and the *HAC2* gene was only expressed in salt-resistant cultivar especially after 7 days of salinity, suggesting that the HAC2 may contribute to salt stress response in resistant sugar beet genotype. This work comprehensively identified sugar beet HATs, providing preliminary knowledge for further studies on epigenetic regulation of abiotic stress response in crops.

### Supplementary Information

Below is the link to the electronic supplementary material.

Supplementary file1 (DOCX 14 KB)

## Data Availability

The HAT data from different plant species presented in this study can be found in National Center for Biotechnology Information (NCBI), Ensembl Plants, The Arabidopsis Information Resource (TAIR), and Phytozome version 13. All data analyzed in this study are already included in the manuscript.

## References

[CR1] Aiese Cigliano R, Sanseverino W, Cremona G, Ercolano MR, Conicella C, Consiglio FM (2013). Genome-wide analysis of histone modifiers in tomato: gaining an insight into their developmental roles. BMC Genomics.

[CR2] Allakhverdiev SI, Sakamoto A, Nishiyama Y, Inaba M, Murata N (2000). Ionic and osmotic effects of NaCl-induced inactivation of photosystems I and II in *Synechococcus* sp. Plant Physiol.

[CR3] Aquea F, Timmermann T, Arce-Johnson P (2010). Analysis of histone acetyltransferase and deacetylase families of Vitis vinifera. Plant Physiol Biochem.

[CR4] Bailey TL, Elkan C (1994). Fitting a mixture model by expectation maximization to discover motifs in biopolymers. Second international conference on intelligent systems for molecular biology, California.

[CR5] Biłas R, Szafran K, Hnatuszko-Konka K, Kononowicz AK (2016). Cis-regulatory elements used to control gene expression in plants. Plant Cell Tiss Organ Cult.

[CR6] Bo H, Jinpu J, An-Yuan G, He Z, Jingchu L, Ge G (2015). GSDS 2.0: an upgraded gene feature visualization server. Bioinformatics.

[CR7] Bolser DM, Staines DM, Perry E, Kersey PJ (2017). Ensembl plants: integrating tools for visualizing, mining, and analyzing plant genomic data. Methods Mol Biol.

[CR8] Bor M, Özdemir F, Türkan I (2003). The effect of salt stress on lipid peroxidation and antioxidants in leaves of sugar beet *Beta*
*vulgaris* L. and wild beet *Beta*
*maritima* L.. Plant Sci.

[CR9] Cai Y, Xu M, Liu J, Zeng H, Song J, Sun B, Chen S, Deng Q, Lei J, Cao B, Chen C, Chen M, Chen K, Chen G, Zhu Z (2022). Genome-wide analysis of histone acetyltransferase and histone deacetylase families and their expression in fruit development and ripening stage of pepper (*Capsicum*
*annuum*). Front Plant Sci.

[CR10] Chao J, Li Z, Sun Y, Aluko OO, Wu X, Wang Q, Liu G (2021). MG2C: a user-friendly online tool for drawing genetic maps. Mol Horticulture.

[CR11] Chen ZJ, Tian L (2007). Roles of dynamic and reversible histone acetylation in plant development and polyploidy. Biochim Biophys Acta.

[CR12] Chinnusamy V, Gong Z, Zhu JK (2008). Abscisic acid-mediated epigenetic processes in plant development and stress responses. J Integr Plant Biol.

[CR13] Clarke N, Hetschkun H, Jones C, Boswell E, Marfaing H, Jackson MB, Black CR (1993). Identification of stress tolerance traits in sugar beet. Interacting stresses on plants in a changing climate.

[CR14] Clements A, Rojas JR, Trievel RC, Wang L, Berger SL, Marmorstein R (1999). Crystal structure of the histone acetyltransferase domain of the human PCAF transcriptional regulator bound to coenzyme A. EMBO J.

[CR15] Darzentas N (2010). Circoletto: visualizing sequence similarity with Circos. Bioinformatics.

[CR16] Dohm JC, LangeHoltgrawe D, Sörensen TR, Borchardt D, Schulz B, Lehrach H, Weisshaar B, Himmelbauer H (2011). Palaeohexaploid ancestry for Caryophyllales inferred from extensive gene-based physical and genetic mapping of the sugar beet genome (*Beta*
*vulgaris*). Plant J.

[CR17] Dohm JC, Minoche AE, Holtgräwe D, Capella-Gutiérrez S, Zakrzewski F, Tafer H, Rupp O, Sörensen TR, Stracke R, Reinhardt R, Goesmann A, Kraft T, Schulz B, Stadler PF, Schmidt T, Gabaldón T, Lehrach H, Weisshaar B, Himmelbauer H (2014). The genome of the recently domesticated crop plant sugar beet (*Beta*
*vulgaris*). Nature.

[CR18] Dong Y, Chen S, Cheng S, Zhou W, Ma Q, Chen Z, Fu CX, Liu X, Zhao YP, Soltis PS, Wong GK, Soltis DE, Xiang QJ (2019). Natural selection and repeated patterns of molecular evolution following allopatric divergence. Elife.

[CR19] Dönnes P, Höglund A (2004). Predicting protein subcellular localization: past, present, and future. Genom Proteom Bioinform.

[CR20] Dunajska-Ordak K, Skorupa-Kłaput M, Kurnik K, Tretyn A, Tyburski J (2014). Cloning and expression analysis of a gene encoding for ascorbate peroxidase and responsive to salt stress in beet (*Beta*
*vulgaris*). Plant Mol Biol Rep.

[CR21] Gao S, Li L, Han X, Liu T, Jin P, Cai L, Xu M, Zhang T, Zhang F, Chen J, Yang J, Zhong K (2021). Genome-wide identification of the histone acetyltransferase gene family in *Triticum*
*aestivum*. BMC Genomics.

[CR22] Gasteiger E, Hoogland C, Gattiker A, Duvaud S, Wilkins MR, Appel RD, Bairoch A, Walker JM (2005). Protein identification and analysis tools on the ExPASy Server. The proteomics protocols handbook.

[CR23] Geng G, Lv C, Stevanato P, Li R, Liu H, Yu L, Wang Y (2019). Transcriptome analysis of salt-sensitive and tolerant genotypes reveals salt-tolerance metabolic pathways in sugar beet. Int J Mol Sci.

[CR24] Geourjon C, Deléage G (1995). SOPMA: significant improvements in protein secondary structure prediction by consensus prediction from multiple alignments. Comput Appl Biosci.

[CR25] Giraudat J, Parcy F, Bertauche N, Gosti F, Leung J, Morris PC, Bouvier-Durand M, Vartanian N (1994). Current advances in abscisic acid action and signalling. Plant Mol Biol.

[CR26] Hawar A, Xiong S, Yang Z, Sun B (2021). Histone acetyltransferase SlGCN5 regulates shoot meristem and flower development in *Solanum*
*lycopersicum*. Front Plant Sci.

[CR27] Hoagland DR, Arnon DI (1950). The water-culture method for growing plants without soil. Circ California Agric Exp Station.

[CR28] Hoffmann CM (2010). Sucrose accumulation in sugar beet under drought stress. J Agron Crop Sci.

[CR29] Horton P, Park KJ, Obayashi T, Fujita N, Harada H, Adams-Collier CJ, Nakai K (2007). WoLF PSORT: protein localization predictor. Nucleic Acids Res.

[CR30] Hussein H-AA, Mekki BB, El-Sadek MEA, El Lateef EE (2019). Effect of L-Ornithine application on improving drought tolerance in sugar beet plants. Heliyon.

[CR31] Ivanova T, Dincheva I, Badjakov I, Iantcheva A (2023). Transcriptional and metabolic profiling of *Arabidopsis*
*thaliana* transgenic plants expressing histone acetyltransferase HAC1 upon the application of abiotic stress-salt and low temperature. Metabolites.

[CR32] Jiangtao C, Yingzhen K, Qian W, Yuhe S, Daping G, Jing L, Guanshan L (2015). Mapgene2chrom, a tool to draw gene physical map based on perl and svg languages. Yi Chuan.

[CR33] Kelley L, Mezulis S, Yates C, Wass MN, Sternberg MJE (2015). The Phyre2 web portal for protein modeling, prediction and analysis. Nat Protoc.

[CR34] Kim JM, To TK, Nishioka T, Seki M (2010). Chromatin regulation functions in plant abiotic stress responses. Plant Cell Environ.

[CR35] Kim JM, Sasaki T, Ueda M, Sako K, Seki M (2015). Chromatin changes in response to drought, salinity, heat, and cold stresses in plants. Front Plant Sci.

[CR36] Kouzarides T (2007). Chromatin modifications and their function. Cell.

[CR37] Krzywinski M, Schein J, Birol I, Connors J, Gascoyne R, Horsman D, Jones SJ, Marra MA (2009). Circos: an information aesthetic for comparative genomics. Genome Res.

[CR38] Latrasse D, Benhamed M, Henry Y, Domenichini S, Kim W, Zhou DX, Delarue M (2008). The MYST histone acetyltransferases are essential for gametophyte development in Arabidopsis. BMC Plant Biol.

[CR39] Lescot M, Déhais P, Thijs G, Marchal K, Moreau Y, Van de Peer Y, Rouzé P, Rombauts S (2002). PlantCARE, a database of plant cis-acting regulatory elements and a portal to tools for in silico analysis of promoter sequences. Nucleic Acids Res.

[CR40] Letunic I, Doerks T, Bork P (2012). SMART 7: recent updates to the protein domain annotation resource. Nucleic Acids Res.

[CR41] Li S, Lin Y-CJ, Wang P, Zhang B, Li M, Chen S, Shi R, Tunlaya-Anukit S, Liu X, Wang Z, Dai X, Yu J, Zhou C, Liu B, Wang JP, Chiang VL, Li W (2018). The AREB1 transcription factor influences histone acetylation to regulate drought responses and tolerance in *Populus*
*trichocarpa*. Plant Cell.

[CR42] Li H, Liu H, Pei X, Chen H, Li X, Wang J, Wang C (2022). Comparative genome-wide analysis and expression profiling of histone acetyltransferases and histone deacetylases involved in the response to drought in wheat. J Plant Growth Regul.

[CR43] Liu X (2017). Application of bioinformatics on protein structure prediction. Destech Trans Comput Sci Eng.

[CR44] Liu X, Luo M, Zhang W, Zhao J, Zhang J, Wu K, Tian L, Duan J (2012) Histone acetyltransferases in rice (*Oryza**sativa* L.): phylogenetic analysis, subcellular localization and expression. BMC Plant Biol. http://www.biomedcentral.com/1471-2229/12/145 10.1186/1471-2229-12-145 PMC3502346 22894565

[CR45] Livak KJ, Schmittgen TD (2001). Analysis of relative gene expression data using real-time quantitative PCR and the 2(-Delta Delta C(T)) Method. Methods.

[CR46] Lv X, Jin Y, Wang Y (2018). De novo transcriptome assembly and identification of salt-responsive genes in sugar beet M14. Comput Biol Chem.

[CR47] Madlung A, Comai L (2004). The effect of stress on genome regulation and structure. Ann Bot.

[CR48] Mai A, Rotili D, Tarantino D, Nebbioso A, Castellano S, Sbardella G, Tini M, Altucci L (2009). Identification of 4-hydroxyquinolines inhibitors of p300/CBP histone acetyltransferases. Bioorg Med Chem Lett.

[CR49] Mao Y, Pavangadkar KA, Thomashow MF, Triezenberg SJ (2006). Physical and functional interactions of Arabidopsis ADA2 transcriptional coactivator proteins with the acetyltransferase GCN5 and with the cold-induced transcription factor CBF1. Biochim Biophys Acta.

[CR50] Nakai K, Horton P (1999). PSORT: a program for detecting sorting signals in proteins and predicting their subcellular localization. Trends Biochem Sci.

[CR51] Pandey R, Müller A, Napoli CA, Selinger DA, Pikaard CS, Richards EJ, Bender J, Mount DW, Jorgensen RA (2002). Analysis of histone acetyltransferase and histone deacetylase families of *Arabidopsis*
*thaliana* suggests functional diversi^®^cation of chromatin modification among multicellular eukaryotes. Nucleic Acids Res.

[CR52] Peng M, Ying P, Liu X, Li C, Xia R, Li J, Zhao M (2017). Genome-wide identification of histone modifiers and their expression patterns during fruit abscission in litchi. Front Plant Sci.

[CR53] Pinheiro C, Ribeiro IC, Reisinger V, Planchon S, Veloso MM, Renaut J, Eichacker L, Ricardo CP (2018). Salinity effect on germination, seedling growth and cotyledon membrane complexes of a Portuguese salt marsh wild beet ecotype. Theor Experiment Plant Physiol.

[CR54] Sahu PP, Pandey G, Sharma N, Puranik S, Muthamilarasan M, Prasad M (2013). Epigenetic mechanisms of plant stress responses and adaptation. Plant Cell Rep.

[CR55] Schmitz RJ, Grotewold E, Stam M (2022). Cis-regulatory sequences in plants: their importance, discovery, and future challenges. Plant Cell.

[CR56] Shahbazian MD, Grunstein M (2007). Functions of site-specific histone acetylation and deacetylation. Annu Rev Biochem.

[CR57] Shu B, Xie Y, Zhang F, Zhang D, Liu C, Wu Q, Luo C (2021). Genome-wide identification of citrus histone acetyltransferase and deacetylase families and their expression in response to arbuscular mycorrhizal fungi and drought. J Plant Interact.

[CR58] Skorupa M, Szczepanek J, Mazur J, Domagalski K, Tretyn A, Tyburski J (2021). Salt stress and salt shock differently affect DNA methylation in salt-responsive genes in sugar beet and its wild, halophytic ancestor. PLoS One.

[CR59] Song H, Ding G, Zhao C, Li Y (2023). Genome-wide identification of B-Box gene family and expression analysis suggest its roles in responses to Cercospora leaf spot in sugar beet (*Beta*
*vulgaris* L.). Genes.

[CR60] Sterner DE, Berger SL (2000). Acetylation of histones and transcription-related factors. Microbiol Mol Biol Rev.

[CR61] Strahl BD, Allis CD (2000). The language of covalent histone modifications. Nature.

[CR62] Tamura K, Stecher G, Kumar S (2021). MEGA11: molecular evolutionary genetics analysis version 11. Mol Biol Evol.

[CR63] Thomas T, Dixon MP, Kueh AJ, Voss AK (2008). Mof (MYST1 or KAT8) is essential for progression of embryonic development past the blastocyst stage and required for normal chromatin architecture. Mol Cell Biol.

[CR64] Wang W, Sun YQ, Li GL, Zhang SY (2019). Genome-wide identification, characterization, and expression patterns of the BZR transcription factor family in sugar beet (*Beta*
*vulgaris* L.). BMC Plant Biol.

[CR65] Waterhouse A, Bertoni M, Bienert S, Studer G, Tauriello G, Gumienny R, Heer FT, de Beer TAP, Rempfer C, Bordoli L, Lepore R, Schwede T (2018). SWISS-MODEL: homology modelling of protein structures and complexes. Nucleic Acids Res.

[CR66] Wedeking R, Mahlein AK, Steiner U, Oerke EC, Goldbach HE, Wimmer MA (2016). Osmotic adjustment of young sugar beets (*Beta*
*vulgaris*) under progressive drought stress and subsequent rewatering assessed by metabolite analysis and infrared thermography. Funct Plant Biol.

[CR67] Williams CJ, Headd JJ, Moriarty NW, Prisant MG, Videau LL, Deis LN, Verma V, Keedy DA, Hintze BJ, Chen VB, Jain S, Lewis SM, Arendall WB, Snoeyink J, Adams PD, Lovell SC, Richardson JS, Richardson DC (2018). MolProbity: more and better reference data for improved all-atom structure validation. Protein Sci.

[CR68] Wu X, Oh MH, Schwarz EM, Larue CT, Sivaguru M, Imai BS, Yau PM, Ort DR, Huber SC (2011). Lysine acetylation is a widespread protein modification for diverse proteins in Arabidopsis. Plant Physiol.

[CR69] Xing G, Jin M, Qu R, Zhang J, Han Y, Han Y, Wang X, Li X, Ma F, Zhao X (2022). Genome-wide investigation of histone acetyltransferase gene family and its responses to biotic and abiotic stress in foxtail millet (*Setaria*
*italica* [L.] P. Beauv). BMC Plant Biol.

[CR70] Xu G, Guo C, Shan H, Kong H (2012). Divergence of duplicate genes in exon–intron structure. Proc Natl Acad Sci.

[CR71] Yang X, Wu G, Wei M, Wang B (2022). Genome-wide identification of BvHAK gene family in sugar beet (*Beta*
*vulgaris*) and their expression analysis under salt treatments. Sheng Wu Gong Cheng Xue Bao.

[CR72] Yolcu S, Ozdemir F, Güler A, Bor M (2016). Histone acetylation influences the transcriptional activation of POX in *Beta*
*vulgaris* L. and *Beta*
*maritima* L. under salt stress. Plant Physiol Biochem.

[CR73] Yolcu S, Alavilli H, Ganesh P, Asif M, Kumar M, Song K (2022). An insight into the abiotic stress responses of cultivated beets (*Beta*
*vulgaris* L.). Plants.

[CR74] Yu CS, Chen YC, Lu CH, Hwang JK (2006). Prediction of protein subcellular localization. Proteins.

[CR75] Yu B, Chen M, Grin I, Ma C (2020). Mechanisms of sugar beet response to biotic and abiotic stresses. Adv Exp Med Biol.

[CR76] Yu Q, Guo Q, Li B, Tan X, Wang L, Li S, Pi Z (2023). Identification of RPD3/HDA1 family genes in sugar beet and response to abiotic stresses. Sugar Tech.

[CR77] Yuan L, Liu X, Luo M, Yang S, Wu K (2013). Involvement of histone modifications in plant abiotic stress responses. J Integr Plant Biol.

[CR78] Zhang Y, Sun T, Liu S, Dong L, Liu C, Song W, Liu J, Gai S (2016). MYC cis-elements in PsMPT promoter is involved in chilling response of *Paeonia*
*suffruticosa*. PLoS ONE.

[CR79] Zhang P, Liu L, Wang X, Wang Z, Zhang H, Chen J, Liu X, Wang Y, Li C (2021). Beneficial effects of exogenous melatonin on overcoming salt stress in sugar beets (*Beta*
*vulgaris* L.). Plants (basel).

[CR80] Zheng M, Liu X, Lin J, Liu X, Wang Z, Xin M, Yao Y, Peng H, Zhou DX, Ni Z, Sun Q, Hu Z (2019). Histone acetyltransferase GCN5 contributes to cell wall integrity and salt stress tolerance by altering the expression of cellulose synthesis genes. Plant J.

