# Supplementary material for: Genome-wide identification, phylogenetic classification of histone acetyltransferase genes, and their expression analysis in sugar beet (Beta vulgaris L.) under salt stress
Source: Planta. 2024 Mar 6;259(4):85. doi: 10.1007/s00425-024-04361-x (PMC10917867; doi:10.1007/s00425-024-04361-x)
Supplement: Supplementary file 1 — Supplementary file1 (DOCX 14 KB) [file 425_2024_4361_MOESM1_ESM.docx]

**Supplementary table**

**Table S1.** Primer sequences and the lengths of PCR products.

| **gene** | **Primer sequence (5’->3’)** | | **Product**  **size (bp)** |
| --- | --- | --- | --- |
| *BvHAG1* | F | GATCTCAAGCATCCCCCTGGC | 123 |
|  | R | TCTTGCCATCAATCTCAAACATCG |  |
| *BvHAG2* | F | TGAGCATATGGTTTGGCTGA | 152 |
|  | R | AAGTGATGCCACCAACAACA |  |
| *BvHAG3* | F | GCTGCTTAACCATGTTGCTG | 151 |
|  | R | TGTTGCCGCTGAATTAACTG |  |
| *BvHAG4* | F | TATGGTGCCTCCTTGGACTC | 161 |
|  | R | TCCCAGCTTCTCTTGTTCGT |  |
| *BvHAF1* | F | GTTGGTGATCGTGAGTCAGTAG | 189 |
|  | R | GACAGTGAGAGATGGTCCCAGT |  |
| *BvHAC1* | F | GGCAAATTAGGGTTTCGTCA | 165 |
|  | R | GCAACCAGCAAGCATAGTGA |  |
| *BvHAC2* | F | TGACCTTGGTAACCGTGTGA | 185 |
|  | R | TTTGCATGAGAGTGGACTGC |  |
| *β-actin* | F | ATCCAGGCCGTTCTTTCTCT | 144 |
|  | R | ACGACCAGCAAGATCCAAAC |  |
